# Supplementary material for: Emulator-based Bayesian inference on non-proportional scintillation models by compton-edge probing
Source: Nat Commun. 2023 Nov 28;14:7790. doi: 10.1038/s41467-023-42574-y (PMC10682496; doi:10.1038/s41467-023-42574-y)
Supplement: Supplementary file 1 — Supplementary Information [file 41467_2023_42574_MOESM1_ESM.pdf]

Supplementary Information for

Emulator-based Bayesian Inference on Non-Proportional Scintillation Models by Compton-Edge Probing

David Breitenmoser[1,2,*], Francesco Cerutti[3], Gernot Butterweck[1], Malgorzata Magdalena Kasprzak[1], Sabine Mayer[1]

[1]Department of Radiation Safety and Security, Paul Scherrer Institute (PSI), Forschungsstrasse 111, Villigen PSI, 5232, Switzerland
[2]Department of Physics, Swiss Federal Institute of Technology (ETH), Otto-Stern-Weg 5, Zurich, 8093, Switzerland
[3]European Organization for Nuclear Research (CERN), Esplanade des Particules 1, Geneva, 1211, Switzerland

[*]Corresponding author: David Breitenmoser (david.breitenmoser@psi.ch, ORCID: 0000-0003-0339-6592)

The PDF includes:

Supplementary Methods S1.1–S1.4
Supplementary Figures S1–S29
Supplementary Tables S1–S5
Supplementary Algorithm S1
Supplementary References

Contents

List of Supplementary Figures **SII**

List of Supplementary Tables **SII**

Supplementary Methods **S1**

S1.1 Adaptive sparse PCE-PCA surrogate model S1
S1.2 PCA-PCE based Hoeffding-Sobol decomposition & Sobol indices S3
S1.3 Uncertainty analysis S6
S1.4 Compton edge shift analysis S8

Supplementary Figures **S13**

Supplementary Tables **S42**

Supplementary Algorithms **S47**

Supplementary References **S48**

List of Supplementary Figures

S1 Markov Chain Monte Carlo trace plots for the sum mode S13
S2 Markov Chain Monte Carlo trace plots for crystal 1 . . . . . S14
S3 Markov Chain Monte Carlo trace plots for crystal 2 . . . . . S15
S4 Markov Chain Monte Carlo trace plots for crystal 3 . . . . . S16
S5 Markov Chain Monte Carlo trace plots for crystal 4 . . . . . S17
S6 Posterior point estimator convergence for the sum mode S18
S7 Posterior point estimator convergence for crystal 1 . . . . . S19
S8 Posterior point estimator convergence for crystal 2 . . . . . S20
S9 Posterior point estimator convergence for crystal 3 . . . . . S21
S10 Posterior point estimator convergence for crystal 4 . . . . . S22
S11 Posterior distribution estimate for crystal 1 . . . . . S23
S12 Posterior distribution estimate for crystal 2 . . . . . S24
S13 Posterior distribution estimate for crystal 3 . . . . . S25
S14 Posterior distribution estimate for crystal 4 . . . . . S26
S15 Compton edge predictions for the individual crystals S27
S16 Spectral comparison of the sum and single mode inversion pipelines S28
S17 Hoeffding-Sobol decomposition for the individual crystals S29
S18 Spectral detector response for ^{57}Co, ^{109}Cd, ^{133}Ba and ^{152}Eu S30
S19 Uncertainty quantification for the ^{60}Co spectral detector response S31
S20 Uncertainty quantification for the ^{88}Y spectral detector response S32
S21 Uncertainty quantification for the ^{137}Cs spectral detector response S33
S22 Uncertainty quantification for the ^{57}Co spectral detector response S34
S23 Uncertainty quantification for the ^{109}Cd spectral detector response S35
S24 Uncertainty quantification for the ^{133}Ba spectral detector response S36
S25 Uncertainty quantification for the ^{152}Eu spectral detector response S37
S26 Compton edge shift analysis for the sum channel S38
S27 Light yield analysis S39
S28 Semi-analytical model results S40
S29 Predicted Compton edge shift for different scintillator sizes S41

List of Supplementary Tables

S1 Prior distribution summary S42
S2 Posterior statistics summary S43
S3 Compton edge domain sensitivity S44
S4 Empirical model summary S45
S5 Material properties for NaI(Tl) scintillator S46

Supplementary Methods

S1.1 Adaptive sparse PCE-PCA surrogate model

Here, based on previous work [1–3], we derive our custom vector-valued adaptive sparse polynomial chaos expansion surrogate model (PCE), which we combine with principal component analysis (PCA).

We start with the PCA model part. Consider our vector-valued model response as a random vector $\mathbf{Y} \in \mathbb{R}^{N\times 1}$ with mean $\boldsymbol{\mu}_{\mathbf{Y}}$, standard deviation $\boldsymbol{\sigma}_{\mathbf{Y}}$ and correlation matrix $\boldsymbol{\Sigma}_{\mathbf{Y}} := \mathrm{corr}\left(\mathbf{Y}\right) = \mathbb{E}\left[\mathbf{Y}^{*}\left(\mathbf{Y}^{*}\right)^{\intercal}\right]$. Note that, in contrast to previous studies [1–3], we standardize our model response $\mathbf{Y}$ with $\mathbf{Y}^{*} := \mathrm{diag}\left(\boldsymbol{\sigma}_{\mathbf{Y}}\right)^{-1}\left(\mathbf{Y} - \boldsymbol{\mu}_{\mathbf{Y}}\right)$ to account for the differences in the variance of the individual response variables. We can then perform an eigenvalue decomposition of the correlation matrix $\boldsymbol{\Sigma}_{\mathbf{Y}}$ with the eigenvalues λ_j and eigenvectors $\boldsymbol{\phi}_j := (\phi_1, \ldots, \phi_N)^{\intercal}$ satisfying $\boldsymbol{\Sigma}_{\mathbf{Y}}\boldsymbol{\phi}_j = \lambda_j\boldsymbol{\phi}_j$ for $j = 1, \ldots, N$. Since $\boldsymbol{\Sigma}_{\mathbf{Y}}$ is symmetric and positive definite, the eigenvectors define an orthonormal basis $\mathbb{R}^N = \mathrm{span}(\{\boldsymbol{\phi}_j\}_{j=1}^{N})$ and we can perform an orthogonal transformation of our random vectors $\mathbf{Y}^{*}$ as follows:

$$\mathbf{Z} = \boldsymbol{\Phi}^{\intercal}\mathbf{Y}^{*} \tag{S1}$$

with the orthonormal matrix $\boldsymbol{\Phi} := (\boldsymbol{\phi}_1, \ldots, \boldsymbol{\phi}_N) \in \mathbb{R}^{N\times N}$, where $\lambda_1 \geq \lambda_2 \geq \ldots \geq \lambda_N$. We call the transformed vectors $\mathbf{Z} := (Z_1, \ldots, Z_N)^{\intercal}$ the principal components of $\mathbf{Y}^{*}$. Once we get the principal components, we can transform them back to the original response variable space with

$$\mathbf{Y} = \boldsymbol{\mu}_{\mathbf{Y}} + \mathrm{diag}\left(\boldsymbol{\sigma}_{\mathbf{Y}}\right)\sum_{j=1}^{N} Z_j\boldsymbol{\phi}_j \tag{S2}$$

To reduce the dimensions of our problem, we retain only N' principal components with the highest variance and thereby approximate our random vector $\mathbf{Y}$ as

$$\mathbf{Y} \approx \boldsymbol{\mu}_{\mathbf{Y}} + \mathrm{diag}\left(\boldsymbol{\sigma}_{\mathbf{Y}}\right)\sum_{j=1}^{N'} Z_j\boldsymbol{\phi}_j \tag{S3}$$

where we choose $N' := \min\{S \in \{1, \ldots, N\} : \sum_{j=1}^{S}\lambda_j / \sum_{j=1}^{N}\lambda_j \geq 1 - \varepsilon_{\mathrm{PCA}}\}$ with a prescribed approximation error $\varepsilon_{\mathrm{PCA}}$.

For the PCE model part, we start again with the polynomial chaos expansion of the model response $\mathcal{M}\left(\mathbf{X}\right)$ with the random input vector $\mathbf{X} \in \mathbb{R}^{M\times 1}$ as described in Eq. 3 in the main study:

$$\mathbf{Y} = \sum_{\boldsymbol{\alpha}\in\mathbb{N}^M} \mathbf{a}_{\boldsymbol{\alpha}}\Psi_{\boldsymbol{\alpha}}\left(\mathbf{X}\right) \tag{S4}$$

where $\mathbf{a}_{\boldsymbol{\alpha}} := (a_{1,\boldsymbol{\alpha}}, \ldots, a_{N,\boldsymbol{\alpha}})^{\intercal} \in \mathbb{R}^{N\times 1}$ are the deterministic expansion coefficients, $\boldsymbol{\alpha} := (\alpha_1, \ldots, \alpha_M)^{\intercal} \in \mathbb{N}^{M\times 1}$ the multi-indices storing the degrees of the univariate polynomials ψ_α and $\Psi_{\boldsymbol{\alpha}}\left(\mathbf{X}\right) := \prod_{i=1}^{M}\psi_{\alpha_i}^{i}\left(X_i\right)$ the multivariate polynomial basis functions, which are orthonormal with respect to the joint probability density function $f_{\mathbf{X}}$ of $\mathbf{X}$, i.e. $\langle\Psi_{\boldsymbol{\alpha}}, \Psi_{\boldsymbol{\beta}}\rangle_{f_{\mathbf{X}}} = \delta_{\boldsymbol{\alpha},\boldsymbol{\beta}}$. For computational purposes, we truncate the PCE series by adopting a truncation set $\mathcal{A}_j$ for the multi-index $\boldsymbol{\alpha}$ of each individual response variable $j = 1, \ldots, N$ resulting in:

$$Y_j \approx \sum_{\boldsymbol{\alpha} \in \mathcal{A}_j} a_{j,\boldsymbol{\alpha}} \Psi_{\boldsymbol{\alpha}} (\mathbf{X}) \tag{S5}$$

For the truncation, we can use a hyperbolic truncation scheme defining the multi-index set as $\mathcal{A}_j \coloneqq \{\boldsymbol{\alpha} \in \mathbb{N}^M : (\sum_{j=1}^M \alpha_j^q)^{1/q} \leq p\}$ with p and q defining the maximum degree for the associated polynomial and the q-norm, respectively.

To reduce the computational burden, we can now combine these results and perform the PCE not in the original response variable space but in the truncated principal component space. For that, we insert Eq. S5 in Eq. S3:

$$\mathbf{Y} \approx \hat{\mathcal{M}} (\mathbf{X}) = \boldsymbol{\mu}_{\mathbf{Y}} + \operatorname{diag}(\boldsymbol{\sigma}_{\mathbf{Y}}) \sum_{j=1}^{N'} \left(\sum_{\boldsymbol{\alpha} \in \mathcal{A}_j} a_{j,\boldsymbol{\alpha}} \Psi_{\boldsymbol{\alpha}} (\mathbf{X}) \right) \boldsymbol{\phi}_j \tag{S6}$$

which we can rearrange by introducing the union set $\mathcal{A}^\star \coloneqq \bigcup_{j=1}^{N'} \mathcal{A}_j$ to:

$$\mathbf{Y} \approx \hat{\mathcal{M}} (\mathbf{X}) = \boldsymbol{\mu}_{\mathbf{Y}} + \operatorname{diag}(\boldsymbol{\sigma}_{\mathbf{Y}}) \sum_{\boldsymbol{\alpha} \in \mathcal{A}^\star} \sum_{j=1}^{N'} a_{j,\boldsymbol{\alpha}} \Psi_{\boldsymbol{\alpha}} (\mathbf{X}) \boldsymbol{\phi}_j \tag{S7}$$

or expressed in a more compact matrix form:

$$\mathbf{Y} \approx \hat{\mathcal{M}} (\mathbf{X}) = \boldsymbol{\mu}_{\mathbf{Y}} + \operatorname{diag}(\boldsymbol{\sigma}_{\mathbf{Y}}) \boldsymbol{\Phi}' \mathbf{A} \boldsymbol{\Psi} (\mathbf{X}) \tag{S8}$$

with the vector $\boldsymbol{\Psi}(\mathbf{X}) \in \mathbb{R}^{\operatorname{card}(\mathcal{A}^\star) \times 1}$ as well as the two matrices $\boldsymbol{\Phi}' \in \mathbb{R}^{N \times N'}$ and $\mathbf{A} \in \mathbb{R}^{N' \times \operatorname{card}(\mathcal{A}^\star)}$ storing the multivariate orthonormal polynomials $\Psi_{\boldsymbol{\alpha}}$, the retained eigenvectors $\boldsymbol{\phi}_j$ and the PCE coefficients $a_{j,\boldsymbol{\alpha}}$, respectively.

For model training, we introduce an experimental design with the input matrix $\boldsymbol{\mathcal{X}} \in \mathbb{R}^{M \times K}$ and response matrix $\boldsymbol{\mathcal{Y}} \in \mathbb{R}^{N \times K}$ for K instances, M input variables and N response variables. For the PCA model, we can use the response matrix $\boldsymbol{\mathcal{Y}}$ to estimate $\boldsymbol{\mu}_{\mathbf{Y}}$, $\boldsymbol{\sigma}_{\mathbf{Y}}$ as well as $\boldsymbol{\Sigma}_{\mathbf{Y}}$:

$$\hat{\boldsymbol{\mu}}_{\mathbf{Y}} = \frac{1}{K} \sum_{k=1}^{K} \mathbf{y}^{(k)} \tag{S9a}$$

$$\hat{\boldsymbol{\sigma}}_{\mathbf{Y}} = \sqrt{\frac{1}{K-1} \sum_{k=1}^{K} \left(\mathbf{y}^{(k)} - \hat{\boldsymbol{\mu}}_{\mathbf{Y}} \right)^2} \tag{S9b}$$

$$\hat{\boldsymbol{\Sigma}}_{\mathbf{Y}} = \frac{1}{K-1} \boldsymbol{\mathcal{Y}}^* (\boldsymbol{\mathcal{Y}}^*)^\intercal \tag{S9c}$$

with $\boldsymbol{\mathcal{Y}}^*$ denoting the standardized response matrix storing the standardized response variables $\mathbf{y}^* \coloneqq \operatorname{diag}(\hat{\boldsymbol{\sigma}}_{\mathbf{Y}}) (\mathbf{y} - \hat{\boldsymbol{\mu}}_{\mathbf{Y}})$, i.e. $\boldsymbol{\mathcal{Y}}^* \coloneqq \left(\mathbf{y}^{*(k)}, \ldots, \mathbf{y}^{*(k)}, \ldots, \mathbf{y}^{*(K)} \right)^\intercal \in \mathbb{R}^{N \times K}$. On the other hand, a rich variety of non-intrusive and sparse methods exist to estimate the PCE coefficient matrix $\mathbf{A}$ using both, the input matrix $\boldsymbol{\mathcal{X}} \in \mathbb{R}^{M \times K}$ and response matrix $\boldsymbol{\mathcal{Y}}$ [4]. In the main study, we chose the least angle regression algorithm [5] due to its high evaluation speed and its high accuracy even for very small experimental designs.

S1.2 PCA-PCE based Hoeffding-Sobol decomposition & Sobol indices

One of the major advantages to use PCE emulators for computational intense simulations is the relation between PCE and the Hoeffding-Sobol decomposition and thereby Sobol indices [6]. For completeness, we repeat here some of the theory already discussed elsewhere [3, 6–8] and derive the PCA-PCE based Sobol indices accounting for the standardization in the PCA discussed in the previous subsection.

We start with the global variance decomposition theory derived by Sobol [8]. It can be shown that for any univariate integrable function $\mathcal{M}(\mathbf{X})$ with M mutually independent random input variables X_i in $\mathcal{D}_{\mathbf{X}}$ and $i = \{1, 2, \ldots, M\}$, there exists a unique functional decomposition, which is often referred to as Hoeffding-Sobol decomposition [8]:

$$\mathcal{M}(\mathbf{X}) = \mathcal{M}_0 + \sum_{i=1}^{M} \mathcal{M}_i(X_i) + \sum_{1 \leq i < j \leq M} \mathcal{M}_{i,j}(X_i, X_j) + \ldots + \mathcal{M}_{1,2,\ldots,M}(X_1, \ldots, X_M) \quad \text{(S10)}$$

where the following two conditions hold:

1. The first term $\mathcal{M}_0$ is constant and equal to the expected value of $\mathcal{M}(\mathbf{x})$:

$$\mathcal{M}_0 = \mathbb{E}[\mathcal{M}(\mathbf{X})] = \int_{\mathcal{D}_{\mathbf{X}}} \mathcal{M}(\mathbf{x})\, \mathrm{d}\mathbf{x} \quad \text{(S11)}$$

2. All the terms in the functional decomposition are orthogonal:

$$\int_{\mathcal{D}_{\mathbf{X}_u}} \mathcal{M}_u(\mathbf{x}_u)\, dx_{i_k} = 0 \quad , \quad 1 \leq k \leq s \quad \text{(S12)}$$

   with u being defined as a subset of indices, i.e. $u \coloneqq \{i_1, \ldots, i_s\} \subset \{1, \ldots, M\}$

Further assuming that the function $\mathcal{M}(\mathbf{X})$ is square-integrable, the functional decomposition in Eq. S10 may be squared and integrated to provide the variance decomposition:

$$V = \sum_{i=1}^{M} V_i + \sum_{1 \leq i < j \leq M} V_{i,j} + \ldots + V_{1,2,\ldots,M} \quad \text{(S13)}$$

with the total variance V and the partial variances V_u defined as:

$$V = \mathrm{Var}[\mathcal{M}(\mathbf{X})] = \int_{\mathcal{D}_{\mathbf{X}}} \mathcal{M}^2(\mathbf{x})\, \mathrm{d}\mathbf{x} - \mathcal{M}_0^2 \quad \text{(S14a)}$$

$$V_u = \mathrm{Var}[\mathcal{M}_u(\mathbf{X}_u)] = \int_{\mathcal{D}_{\mathbf{X}_u}} \mathcal{M}_u^2(\mathbf{x}_u)\, \mathrm{d}\mathbf{x}_u \quad \text{(S14b)}$$

Based on these results, Sobol indices S_u can be defined as a natural global sensitivity measure of $\mathcal{M}(\mathbf{X})$ on the input variables $\mathbf{X}_u$:

$$S_u \coloneqq \frac{V_u}{V} \quad \text{(S15)}$$

Consequently, S_u represents the relative contribution of the set of variables u to the total variance V. First order indices S_i indicate the influence of X_i alone, whereas the higher order indices quantify possible interactions or mixed influences between multiple variables. In addition, we can also define the total Sobol index S_i^T to evaluate the total effect of an input parameter X_i on $\mathcal{M}(\mathbf{X})$:

$$S_i^T \coloneqq \frac{1}{V} \sum_{u \supset i} V_u \tag{S16}$$

As a result, S_i^T includes not only the effect of X_i alone but in addition the effect induced by all interactions between X_i and the other variables. This is also the reason, why the sum of the total Sobol indices $\sum_i S_i^T$ can in fact exceed 1. As an example, if we have an interaction between the variables X_1 and X_2, their interaction effect on $\mathcal{M}$ is counted twice, once in S_1^T and another time in S_2^T. This example in mind, it is easy to see that the peaks in Fig. 3(e) in the main study highlight regions, where the interaction terms between the individual variables significantly contribute to the total effect on $\mathcal{M}$. We have to add that for our study, the absolute values of S_i^T are of less importance. We are more interested in the relative size of S_i^T, because the comparison of these values allows us to draw conclusions about the relative importance of the corresponding variables X_i for the model response $\mathcal{M}(\mathbf{X})$. As shown by [6], S_i^T can also be computed as:

$$\begin{aligned} S_i^T &= 1 - S_{\sim i} && \text{(S17a)} \\ &= 1 - \frac{\mathrm{Var}_{X_{\sim i}}\left[\mathbb{E}_{X_i}\left[\mathcal{M}(\mathbf{X})\right]\right]}{\mathrm{Var}\left[\mathcal{M}(\mathbf{X})\right]} && \text{(S17b)} \end{aligned}$$

where we use $\sim i$ to denote a set of indices, which do not include i, i.e. $S_{\sim i} = S_v$ with $v = \{1, \ldots, i-1, i+1, \ldots, M\}$.

Suppose now that we have a PCA-PCE surrogate model to emulate the vector-valued model response $\mathbf{Y} = \mathcal{M}(\mathbf{X})$ with the random input vector $\mathbf{X} \in \mathbb{R}^{M \times 1}$ and random response vector $\mathbf{Y} \in \mathbb{R}^{N \times 1}$. To derive the $S_{i,k}^T$ for each response variable $k \in \{1, 2, \ldots, N\}$, we start with $\mathrm{Var}_{X_{\sim i}}\left[\mathbb{E}_{X_i}\left[Y_k\right]\right]$ from Eq. S17b by replacing Y_k with the kth component of Eq. S8:

$$\begin{aligned} \mathrm{Var}_{X_{\sim i}}\left[\mathbb{E}_{X_i}\left[Y_k\right]\right] &= \mathbb{E}_{X_{\sim i}}\left[\left(\mathbb{E}_{X_i}\left[Y_k\right]\right)^2\right] - \left(\mathbb{E}_{X}\left[Y_k\right]\right)^2 && \text{(S18a)} \\ &= \mathbb{E}_{X_{\sim i}}\left[\left(\mathbb{E}_{X_i}\left[\mu_{Y_k} + \sigma_{Y_k} \boldsymbol{\phi}_k^{\text{row}} \mathbf{A} \boldsymbol{\Psi}(\mathbf{X})\right]\right)^2\right] - \mu_{Y_k}^2 && \text{(S18b)} \end{aligned}$$

where we used $\boldsymbol{\phi}_k^{\text{row}} \coloneqq (\phi_{k1}, \ldots, \phi_{kN'})$. We can simplify this expression by expanding the first term and considering that the expectation vanishes for all principal components, i.e. $\mathbb{E}\left[\mathbf{A}\boldsymbol{\Psi}(\mathbf{X})\right] = 0$:

$$\begin{aligned} \mathrm{Var}_{X_{\sim i}}\left[\mathbb{E}_{X_i}\left[Y_k\right]\right] &= \mathbb{E}_{X_{\sim i}}\left[\left(\sigma_{Y_k} \boldsymbol{\phi}_k^{\text{row}} \mathbf{A} \mathbb{E}\left[\boldsymbol{\Psi}(\mathbf{X})\right]\right)^2\right] && \text{(S19a)} \\ &= \mathbb{E}_{X_{\sim i}}\left[\left(\sum_{\boldsymbol{\alpha} \in \mathcal{A}^\star} \sum_{j=1}^{N'} \sigma_{Y_k} \phi_{kj} a_{j,\boldsymbol{\alpha}} \mathbb{E}\left[\boldsymbol{\Psi}(\mathbf{X})\right]\right)^2\right] && \text{(S19b)} \end{aligned}$$

As shown by [3], due to the orthonormality of the polynomial basis $\{\Psi_{\boldsymbol{\alpha}}\}_{\boldsymbol{\alpha}\in\mathcal{A}^\star}$, we can further simplify Eq. S19b resulting in:

$$\mathrm{Var}_{X_{\sim i}}\left[\mathbb{E}_{X_i}\left[Y_k\right]\right] = \sigma_{Y_k}^2 \sum_{\boldsymbol{\alpha}\in\mathcal{A}^\star_{i=0}} \left(\sum_{j=1}^{N'} \phi_{kj} a_{j,\boldsymbol{\alpha}}\right)^2 \tag{S20}$$

with the subset $\mathcal{A}^\star_{i=0} \coloneqq \{\boldsymbol{\alpha} \in \mathcal{A}^\star \mid \alpha_i = 0\}$. Using these results, we can compute the total variance with:

$$\mathrm{Var}\left[Y_k\right] = \sigma_{Y_k}^2 \sum_{\boldsymbol{\alpha}\in\mathcal{A}^\star} \left(\sum_{j=1}^{N'} \phi_{kj} a_{j,\boldsymbol{\alpha}}\right)^2 \tag{S21}$$

In the end, we get the total PCE-PCA based Sobol index $S^T_{i,k}$ for the input variable i and the response variable k by inserting Eq. S20 and Eq. S21 into Eq. S17b:

$$S^T_{i,k} = 1 - \frac{\sum_{\boldsymbol{\alpha}\in\mathcal{A}^\star_{i=0}} \left(\sum_{j=1}^{N'} \phi_{kj}\, a_{j,\boldsymbol{\alpha}}\right)^2}{\sum_{\boldsymbol{\alpha}\in\mathcal{A}^\star} \left(\sum_{j=1}^{N'} \phi_{kj}\, a_{j,\boldsymbol{\alpha}}\right)^2} \tag{S22}$$

S1.3 Uncertainty analysis

For completeness, we repeat here the uncertainty analysis pipeline adopted for the measured and simulated pulse-height spectra and highlight some changes to [9].

For the radiation measurements, the statistical uncertainty of the net count rate spectra $c_{\mathrm{exp},k}$ characterized by the standard deviation was computed adopting a probabilistic Poisson model [10]:

$$\sigma_{\mathrm{pois,exp},k} = \sqrt{\frac{C_{\mathrm{gr},k}}{t_{\mathrm{gr}}^2} + \frac{C_{\mathrm{bg},k}}{t_{\mathrm{bg}}^2}} \tag{S23}$$

where $C_{\mathrm{gr},k}$ and $C_{\mathrm{bg},k}$ are the gross and background counts in channel k together with the gross and background measurement live times t_{gr} and t_{bg}, respectively. The small statistical uncertainty in the live time measurement is neglected. To compute the source activity A as a function of the measurement date t, we use the fundamental exponential law of decay, i.e. $A = A_0 \cdot 2^{-\Delta t/t_{1/2}}$ [10]. The uncertainty induced by the source activity A normalization is quantified using the standard error propagation methodology for independent variables [11]:

$$\sigma_A = \sigma_{A_0} \cdot 2^{-\Delta t/t_{1/2}} \tag{S24}$$

with the reference activity A_0 and associated uncertainty σ_{A_0} provided by the vendor, the source half life $t_{1/2}$ [12] as well as the time difference $\Delta t = t - t_0$ between the reference date t_0 and the measurement date t. Contributions of the uncertainties in $t_{1/2}$ and Δt to σ_A are found to be less than 1% for all performed measurements and are therefore neglected. We then summarize the total experimental uncertainty as follows [11]:

$$\sigma_{\mathrm{tot,exp},k} = \sqrt{\left(\frac{\sigma_{\mathrm{pois,exp},k}}{A}\right)^2 + \left(\frac{c_{\mathrm{exp},k}}{A} \cdot \sigma_A\right)^2} \tag{S25}$$

For the simulations, we computed the statistical uncertainty of the net count rate spectrum $c_{\mathrm{sim},k}$ characterized by the standard deviation as follows [10]:

$$\sigma_{\mathrm{stat,sim},k} = \sqrt{\frac{1}{N_{\mathrm{pr}}\left(N_{\mathrm{pr}} - 1\right)} \cdot \left[\left(N_{\mathrm{pr}} - N_{\mathrm{dep}}\right) \cdot c_{\mathrm{sim},k}^2 + \sum_{l=1}^{N_{\mathrm{dep}}} \left(c_{\mathrm{sim},kl} - c_{\mathrm{sim},k}\right)^2\right]} \tag{S26}$$

where $c_{\mathrm{sim},kl}$ are the individual broadened energy deposition events in the detector channel k, N_{dep} the number of recorded events and N_{pr} the number of simulated primaries. It is good practice in Monte Carlo studies to report not only the estimated uncertainty in the sample mean $c_{\mathrm{sim},k}$ using the sample standard deviation $\sigma_{\mathrm{stat,sim},k}$ but also the so called variance of the sample variance VOV_k for the detector channel k to quantify the statistical uncertainty in $\sigma_{\mathrm{stat,sim},k}^2$ itself [13]:

$$\mathrm{VOV}_k = \frac{\mathrm{Var}\left(\sigma_{\mathrm{stat,sim},k}^2\right)}{\sigma_{\mathrm{stat,sim},k}^4} = \frac{\left(N_{\mathrm{pr}} - N_{\mathrm{dep}}\right) \cdot c_{\mathrm{sim},k}^4 + \sum_{l=1}^{N_{\mathrm{dep}}} \left(c_{\mathrm{sim},kl} - c_{\mathrm{sim},k}\right)^4}{\left[\left(N_{\mathrm{pr}} - N_{\mathrm{dep}}\right) \cdot c_{\mathrm{sim},k}^2 + \sum_{l=1}^{N_{\mathrm{dep}}} \left(c_{\mathrm{sim},kl} - c_{\mathrm{sim},k}\right)^2\right]^2} - \frac{1}{N_{\mathrm{pr}}} \tag{S27}$$

The propagation of the systematic uncertainties for the simulated detector response was performed by the Monte Carlo sampling technique. We considered the same model parameters for the uncertainty propagation as in [9]. These parameters are the energy calibration factor $D_1\left[\mathrm{keV}^{-1}\right]$ as well as the empirical resolution parameters $B_1\,[-]$ and $B_2\,[-]$. However, we adapted the marginal distributions by introducing truncated normal distributions as summarized in Table S4. In addition, we accounted for the statistical dependence of the model parameters B_1 and B_2 by correlated sampling using the Gaussian copula $\mathcal{C}_\mathcal{N}$ [14]:

$$\{B_1^*, B_2\} \sim \mathcal{C}_\mathcal{N}\left(F_{B_1^*}\left(b_1^*\right), F_{B_2}\left(b_2\right);\, \mathbf{R}\right) \tag{S28a}$$

$$\sim \Phi_2\left(\Phi^{-1}\left(F_{B_1^*}\left(b_1^*\right)\right), \Phi^{-1}\left(F_{B_2}\left(b_2\right)\right);\, \mathbf{R}\right) \tag{S28b}$$

with the log-transformed variable $B_1^* \coloneqq \log\left(B_1\right)$, the linear correlation matrix $\mathbf{R}$ obtained by the regression analysis, the marginal distribution functions F provided in Table S4, the bivariate Gaussian distribution function Φ_2 associated with the Gaussian copula $\mathcal{C}_\mathcal{N}$ and the inverse cumulative distribution function of the standard normal distribution Φ^{-1}, respectively. The energy calibration factor D_1 is sampled independently according to the corresponding marginal as in [9]. For more details and relevant literature on the copula theory, the reader is referred to [15, 16].

The $N_{\mathrm{MC}} \in \mathbb{N}_{>1}$ independently drawn input samples $\boldsymbol{\mathcal{X}}_{\mathrm{MC}} = (\mathbf{x}^{(1)}, ..., \mathbf{x}^{(m)}, ...\mathbf{x}^{(N_{\mathrm{MC}})})^\intercal$ from the probabilistic input model with $\mathbf{X} \coloneqq (D_1, B_1, B_2)^\intercal$ are then propagated through the post-processing pipeline described in [9] to obtain the corresponding spectral count rate samples $\boldsymbol{\mathcal{Y}}_{\mathrm{MC}} = (c_{\mathrm{sim},k}^{(1)}, ..., c_{\mathrm{sim},k}^{(m)}, ..., c_{\mathrm{sim},k}^{(N_{\mathrm{MC}})})^\intercal$ with $k \in \{1, ..., 1024\}$. These samples can then be used to compute the sample standard deviation $\sigma_{\mathrm{sys,sim},k}$ similar to Eq. S9b and thereby quantify the systematic uncertainty with respect to the empirical model parameters D_1, B_1 and B_2. The total uncertainty characterized by the sample standard deviation can be summarized in the same way as for the experimental uncertainty [11]:

$$\sigma_{\mathrm{tot,sim},k} = \sqrt{\sigma_{\mathrm{stat,sim},k}^2 + \sigma_{\mathrm{sys,sim},k}^2} \tag{S29}$$

S1.4 Compton edge shift analysis

To better understand the nature of the Compton edge shift utilized in our study, we quantify here the spectral shift between the measured Compton edge energy and the theoretical value according to the Compton scattering theory (cf. Eq. 2 in the main study). Because the measured Compton edges are obscured by the finite spectral resolution, quantification of the exact position in the measured pulse-height spectrum would require additional coincidence measurements [17, 18]. Therefore, similar to a previous study [19], we use an alternative approach by quantifying the spectral shift between the already available Monte Carlo simulations with a proportional scintillation response and the measured pulse-height spectra. It is important to add that, based on the findings reported in the main study as well as due to the improved signal-to-noise ratio, we focus our investigation here on the sum channel.

In a first step, we determine the inflection points as a characteristic measure of the corresponding Compton edges using spline regression [20]. We then compute the Compton edge shift as the spectral difference between the determined inflection points for the measured and simulated spectra. We apply this method to different Compton edges, i.e. 477.334(3) keV associated with the ^{137}Cs emission line at 661.657(3) keV, 699.133(3) keV associated with the ^{88}Y emission line at 898.042(3) keV, 963.419(3) keV associated with the ^{60}Co emission line at 1173.228(3) keV and 1611.77(1) keV associated with the ^{88}Y emission line at 1836.063(3) keV. Moreover, we perform Monte Carlo based uncertainty quantification by systematically propagating the uncertainty in the hyperparamters for the individual spline regression models.

In the Fig. S26, we present the results of our Compton edge shift analysis for the sum channel. In general, we can identify a consistent trend in the shift, i.e. an enhanced Compton edge shift toward smaller spectral energies with increasing Compton edge energy. As discussed in the main study, our NPSM predicts the Compton edge shift for all analyzed Compton edges with high accuracy. However, because our approach is based on complex Monte Carlo simulations, interpretability of the results and their connection to the various underlying physical processes is challenging. Therefore, to improve our understanding of the relationship between the NPSM and the resulting Compton edge shift, we develop here a simplified semi-analytical model.

We start by deriving the light yield function $\mathcal{L}$ as a function of the initial electron kinetic energy E_k by integrating Eq. 1 from the main study from E_k down to the mean excitation energy I:

$$\mathcal{L}\left(E_\mathrm{k}\right) = \int_I^{E_\mathrm{k}} L\left(dE/ds\right) dE_\mathrm{k}' \tag{S30}$$

To compute the integral in Eq. S30, we first need a model to describe the differential energy loss dE per differential path length ds as a function of the kinetic electron energy E_k. Similar to Payne and his co-workers [21, 22], we apply a modified Bethe-Bloch model derived by Joy and Luo [23] to described dE/ds as a function of E_k in units of eV Å^{-1}:

$$dE/ds\left(E_\mathrm{k}\right) = 785 \frac{\rho Z}{A E_\mathrm{k}} \log\left[\frac{1.166\left(E_\mathrm{k} + cI\right)}{I}\right] \tag{S31}$$

with the scintillator related mass density ρ, the atomic number Z and the molecular weight A. In accordance with the results obtained by Payne and his co-workers, we fix the stopping power correction factor c in Eq. S31 to $c = 2.8$. To account for radiative losses as well as relativistic effects at higher energies, we combine Joy's model with the ESTAR database [24]. We also consulted the

ESTAR database for all material related properties of NaI(Tl) (cf. Table S5). The resulting total stopping power model together with the individual model components are shown in Fig. S27(a) for NaI(Tl).

We then combine the derived stopping power model with the light yield $L\left(dE/ds\right)$ to perform the integration in Eq. S30. For the model parameters in $L\left(dE/ds\right)$, i.e. $\eta_{e/h}$, $dE/ds\mid_{\mathrm{Ons}}$, $dE/ds\mid_{\mathrm{Trap}}$ and $dE/ds\mid_{\mathrm{Birks}}$, we applied the maximum a posteriori (MAP) probability point estimates to compute a mean light yield function as well as the full set of posterior samples to derive the corresponding credible intervals for both, the sum channel and the individual crystals associated with the sum and single mode inversion pipelines, respectively. The resulting relative light yield functions $\mathcal{L}\left(E_{\mathrm{k}}\right)/E_{\mathrm{k}}$ are shown in Fig. S27(b). The characteristic shape of these relative light yield curves in Fig. S27(b) has been extensively documented by numerous previous empirical studies [21, 22, 25–28], illustrating an increase in light yield with increasing energy for $E_{\mathrm{k}} \ll 10\,\mathrm{keV}$, a prominent peak around $10\,\mathrm{keV}$, followed by a subsequent decrease in yield for higher energies.

After successfully deriving the light yield function $\mathcal{L}$ as a function of the kinetic electron energy E_{k}, we now continue by investigating the connection between the observed negative Compton edge shift and the non-proportional nature of this light yield function. For an ideal detector with a proportional scintillation response, we would have a constant relative light yield function:

$$\frac{\mathcal{L}\left(E_{\mathrm{k}}\right)}{E_{\mathrm{k}}} = \mathrm{const.} \tag{S32}$$

So, we can easily see that for a detector with a non-proportional scintillation light yield function, we get a spectral shift ΔE, if we convert the produced scintillation light of an electron with energy $E_{\mathrm{k},1}$ at a different energy $E_{\mathrm{k},2}$:

$$\Delta E = \frac{\mathcal{L}\left(E_{\mathrm{k},1}\right)}{\mathcal{L}\left(E_{\mathrm{k},2}\right)/E_{\mathrm{k},2}} - \frac{\mathcal{L}\left(E_{\mathrm{k},1}\right)}{\mathcal{L}\left(E_{\mathrm{k},1}\right)/E_{\mathrm{k},1}} \tag{S33a}$$

$$= \frac{\mathcal{L}\left(E_{\mathrm{k},1}\right)}{\mathcal{L}\left(E_{\mathrm{k},2}\right)}E_{\mathrm{k},2} - E_{\mathrm{k},1} \tag{S33b}$$

It is important to add that in our analysis, we implicitly assume a continuous deceleration of the involved electrons, starting from their initial kinetic energy E_{k} and progressing down to the scintillator specific excitation energy I. In particular. we neglect electrons escaping from the scintillator. From Eq. S33b we can conclude that this shift ΔE will be positive for $\mathcal{L}\left(E_{\mathrm{k},1}\right)/E_{\mathrm{k},1} > \mathcal{L}\left(E_{\mathrm{k},2}\right)/E_{\mathrm{k},2}$ and negative for $\mathcal{L}\left(E_{\mathrm{k},1}\right)/E_{\mathrm{k},1} < \mathcal{L}\left(E_{\mathrm{k},2}\right)/E_{\mathrm{k},2}$. Moreover, with an increase in the relative difference $|1 - [\mathcal{L}\left(E_{\mathrm{k},1}\right)/E_{\mathrm{k},1}] / [\mathcal{L}\left(E_{\mathrm{k},2}\right)/E_{\mathrm{k},2}]|$, we expect a proportional increase in the magnitude of the spectral shift.

We can now use these insights for our Compton edge shift analysis. In gamma-ray spectrometry with inorganic scintillators, the energy calibration is typically performed using full energy peaks (FEPs), sometimes also called photopeaks [9, 10]. In other words, to relate the photon energy with the generated scintillation photons, we use the relative light yield function $\sum_{j=1}^{N_{e^-}} \mathcal{L}(E_{\mathrm{k},j}^{\mathrm{FEP}}) / \sum_{j=1}^{N_{e^-}} E_{\mathrm{k},j}^{\mathrm{FEP}}$ for $j = \{1, \ldots, N_{e^-}\}$ electrons with kinetic energy $E_{\mathrm{k},j}$ generated in the scintillator leading subsequently to a FEP. Consequently, in order to compute the Compton edge shift for inorganic scintillators using our model in Eq. S33b, we need to investigate the light

yield for both Compton edge (CE) and FEP events, more specifically the integrated light yield for all electrons generated during these events.

We start with the CE events: As already discussed in the main study, in a CE event, a photon enters the scintillator, undergoes a single Compton scattering (COM) event with a deflection angle of 180°, i.e. full back-scattering, and subsequently escapes the scintillator. During this interaction, some of the photon's energy gets transferred to a single atomic electron. Neglecting Doppler broadening and atomic shell effects [29, 30], the transferred energy $E_{\mathrm{k}}^{\mathrm{CE}}$ is equivalent to the CE energy discussed in Eq. 2 in the main study, i.e.:

$$E_{\mathrm{k}}^{\mathrm{CE}} = E_{\gamma}^{0}\left(1 - \frac{1}{1 + \frac{2E_{\gamma}^{0}}{m_e c^2}}\right) \tag{S34}$$

with E_{γ}^{0} being the initial photon energy and $m_e c^2$ the energy equivalent electron mass. Because only one COM event takes place with a deterministic energy transfer, the light yield for a CE event can easily be calculated as follows:

$$\mathcal{L}\left(E_{\mathrm{k}}^{\mathrm{CE}}\right) = \mathcal{L}\left[E_{\gamma}^{0}\left(1 - \frac{1}{1 + \frac{2E_{\gamma}^{0}}{m_e c^2}}\right)\right] \tag{S35}$$

FEP events on the other hand are more complex because they involve a variable number of COM events with a subsequent photoelectric absorption (PE) of the photon in the scintillator. For simplicity, we neglect again Doppler broadening as well as atomic shell effects and consider only secondary electrons generated during COM and PE events. In particular, we neglect fluorescence photons and Auger electrons. Using these simplifications, we can calculate the light yield for a FEP event involving $j = \{1, \ldots, N_{e^-}\}$ electrons with kinetic energy $E_{\mathrm{k},j}$ as a sequence of N_{COM} COM events followed by a single PE event:

$$\sum_{j=1}^{N_{e-}} \mathcal{L}\left(E_{\mathrm{k},j}^{\mathrm{FEP}}\right) = \mathcal{L}\left(E_{\mathrm{k}}^{\mathrm{PE}}\right) + \sum_{i=1}^{N_{\mathrm{COM}}} \mathcal{L}\left(E_{\mathrm{k},i}^{\mathrm{COM}}\right) \tag{S36a}$$

$$= \mathcal{L}\left(E_{\gamma}^{N_{\mathrm{COM}}}\right) + \sum_{i=1}^{N_{\mathrm{COM}}} \mathcal{L}\left(E_{\gamma}^{i-1} - E_{\gamma}^{i}\right) \tag{S36b}$$

where we denote with $E_{\mathrm{k},j}^{\mathrm{PE}}$ and $E_{\mathrm{k},j}^{\mathrm{COM}}$ the kinetic energies of the electrons generated during PE and COM events, respectively, and with E_{γ}^{i} the photon energy after i subsequent COM events. Both, the number of COM events (N_{COM}) as well as the transferred energy in these COM events ($E_{\gamma}^{i-1} - E_{\gamma}^{i}$) are linked in a complex stochastic process and depend on the photon energy as well as the properties of the scintillator [29–31]. To estimate these variables for our specific detector system, we apply once again Monte Carlo methods. More specifically, we adopted the multi-purpose Monte Carlo code `FLUKA` with the same physics settings as described in the main study [32]. For the semi-analytical model described here, the mass model only included the scintillation crystal embedded in a vacuum environment. To estimate the scintillator response, we irradiated the mass model with an isotropic and uniform monoenergetic photon flux of energy E_{γ}^{0} using the `FLOOD`

mode with the BEAMPOSit card. We repeated these simulations for 31 different photon energies E_γ^0 in the spectral range $[500\,,2000]$ keV with a spacing of 50 keV. To score N_{COM} as well as E_γ^i, we applied the user routine mgdraw.

In Fig. S28(a), we present the probability density for the scored number of COM events before absorption (N_{COM}) as a function of the photon energy E_γ^0 in a prismatic NaI(Tl) scintillator with dimensions 10.2 cm × 10.2 cm × 40.6 cm, i.e. the same crystal size as for our detector system used in the main study. In line with previous results [33], we find a moderate increase of the mean number of COM events with increasing photon energy ranging from 1.4 at 500 keV up to 2.3 at 2000 keV. Combining these results together with Eq. S35 and Eq. S36b, we can now compute the spectral Compton edge shift according to Eq. S33b as follows:

$$\Delta E = E_\gamma^0 \left\{ \frac{\mathcal{L}\left[E_\gamma^0\left(1-\frac{1}{1+\frac{2E_\gamma^0}{m_e c^2}}\right)\right]}{\mathcal{L}\left(E_\gamma^{N_{\mathrm{COM}}}\right)+\sum_{i=1}^{N_{\mathrm{COM}}}\mathcal{L}\left(E_\gamma^{i-1}-E_\gamma^{i}\right)} - 1 + \frac{1}{1+\frac{2E_\gamma^0}{m_e c^2}} \right\} \tag{S37}$$

where we used the fact that for a FEP event in our simplified framework, the following special relationship holds: $\sum_{j=1}^{N_{e^-}} E_{\mathrm{k},j}^{\mathrm{FEP}} = E_\gamma^0$.

In Fig. S28(b), we show the resulting negative Compton edge shift $-\Delta E$ as a function of the photon energy E_γ^0 for the same prismatic NaI(Tl) scintillator. In general, we find a good agreement between the predictions of our simplified semi-analytical model and the experimental results. Deviations can be attributed to the various simplifications and assumptions made during model derivation, e.g. Landau fluctuations, Doppler broadening, atomic shell effects, detector cross-talk, neglected secondary particles such as fluorescence photons and Auger electrons or escaping electrons.

By discriminating the predicted spectral shift for different number of COM events N_{COM}, we can gain also some further insights in the underlying physics. First, we find a pronounced increase in the Compton edge shift $|\Delta E|$, both for an increase in the photon energy E_γ^0 as well as for an increase in the number of COM events N_{COM}. This is in line with our predictions discussed above, i.e. we expect a proportional increase in the magnitude of the spectral shift for an increase in the relative difference $|1-\mathcal{L}(\beta E_\gamma^0)/[\beta\sum_{j=1}^{N_{e^-}}\mathcal{L}(E_{\mathrm{k},j}^{\mathrm{FEP}})]|$ with $\beta := 1-1/[1+2E_\gamma^0/(m_e c^2)]$. It is now easy to see that, due to the decreasing trend in $\mathcal{L}$ for higher energies (cf. Fig. S27(b)), the relative difference will increase for an increase in E_k and subsequently E_γ^0. Furthermore, we find that the relative light yield for CE events is on average smaller than for FEP events with $E_\gamma^0 \in [500\,,2000]$ keV, i.e. $\mathcal{L}(\beta E_\gamma^0)/\beta < \langle\sum_{j=1}^{N_{e^-}}\mathcal{L}(E_{\mathrm{k},j}^{\mathrm{FEP}})\rangle$. This explains the negative sign for the spectral shift observed by our NaI(Tl) detector system.

With this newly derived semi-analytical model, we have now also a tool to investigate the relation between the negative Compton edge shift and the size of a scintillation crystal. In Fig. S29(b), we present the predicted mean Compton edge shift as a function of the photon energy E_γ^0 alongside the relation between N_{COM} and E_γ^0. We find a pronounced and consistent increase in the negative Compton edge shift for an increase in crystal size over the entire spectral domain $[500\,,2000]$ keV. From our semi-analytical model and the results in Fig. S29(a), it is evident that this trend can be explained by the increase in N_{COM} for bigger scintillation crystals.

In summary, the semi-analytical model derived in this section cannot only successfully predict the trends and sign of the Compton edge shift with increasing photon energy E_γ^0, but it can also be used to investigate the relation between crystal size and Compton edge shift and thereby supports the interpretation of the results and findings in the main study obtained by high-fidelity Monte Carlo simulations.

Supplementary Figures

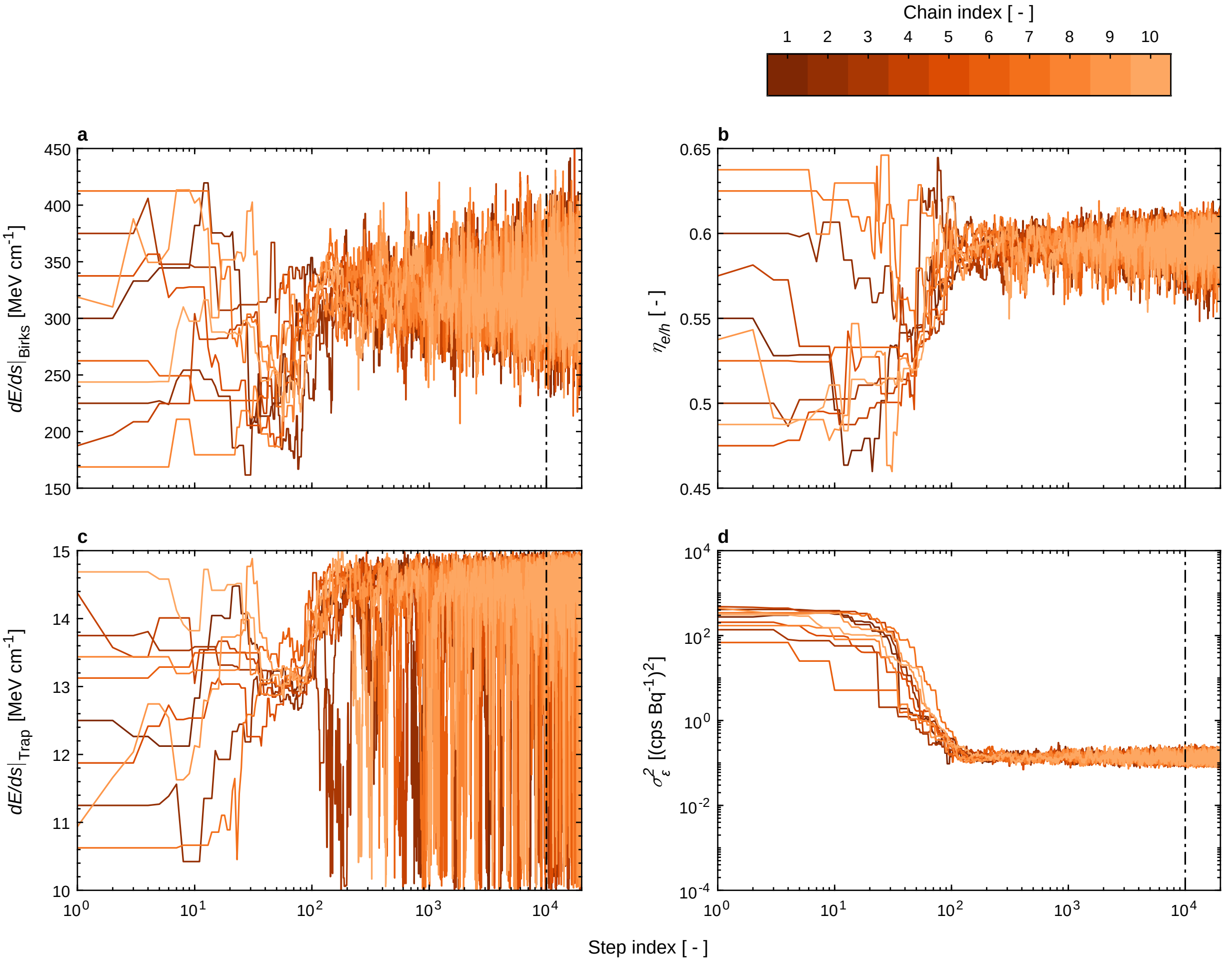

Fig. S1: **Markov Chain Monte Carlo trace plots for the sum mode.** These graphs show the sample values of the Markov Chain Monte Carlo algorithm [34] for each individual Markov chain and model parameter resulting from the sum mode inversion pipeline applied to the sum channel: **a** The Birks related stopping power parameter $dE/ds\ |_{\mathrm{Birks}}$. **b** The free carrier fraction $\eta_{e/h}$. **c** The trapping related stopping power parameter $dE/ds\ |_{\mathrm{Trap}}$. **d** The discrepancy model variance σ_ε^2. In addition, the burn-in threshold is highlighted as a dashed-dotted black line in each graph.

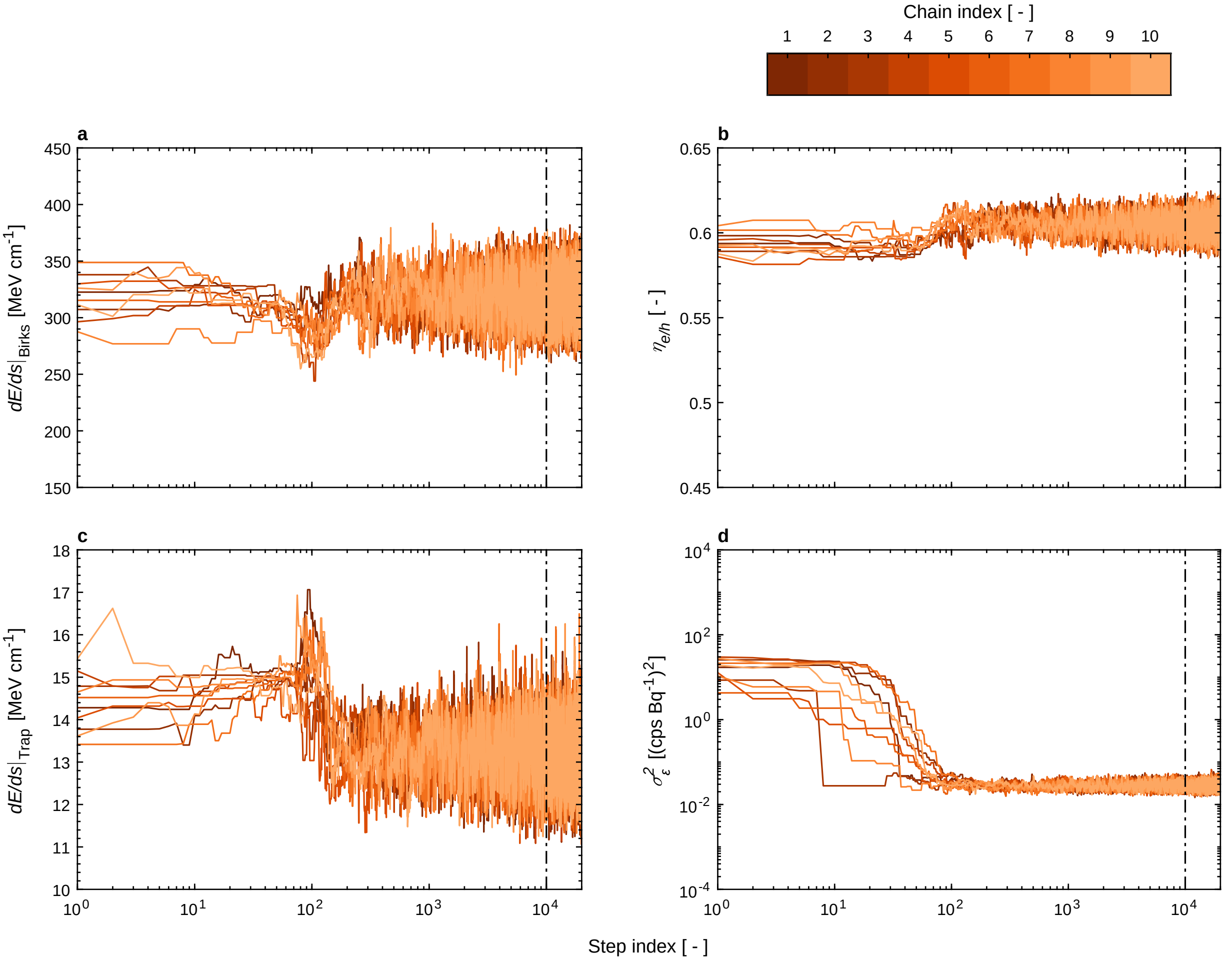

Fig. S2: **Markov Chain Monte Carlo trace plots for crystal 1.** These graphs show the sample values of the Markov Chain Monte Carlo algorithm [34] for each individual Markov chain and model parameter resulting from the single mode inversion pipeline applied to the scintillation crystal 1: **a** The Birks related stopping power parameter $dE/ds\mid_{\text{Birks}}$. **b** The free carrier fraction $\eta_{e/h}$. **c** The trapping related stopping power parameter $dE/ds\mid_{\text{Trap}}$. **d** The discrepancy model variance σ_ε^2. In addition, the burn-in threshold is highlighted as a dashed-dotted black line in each graph.

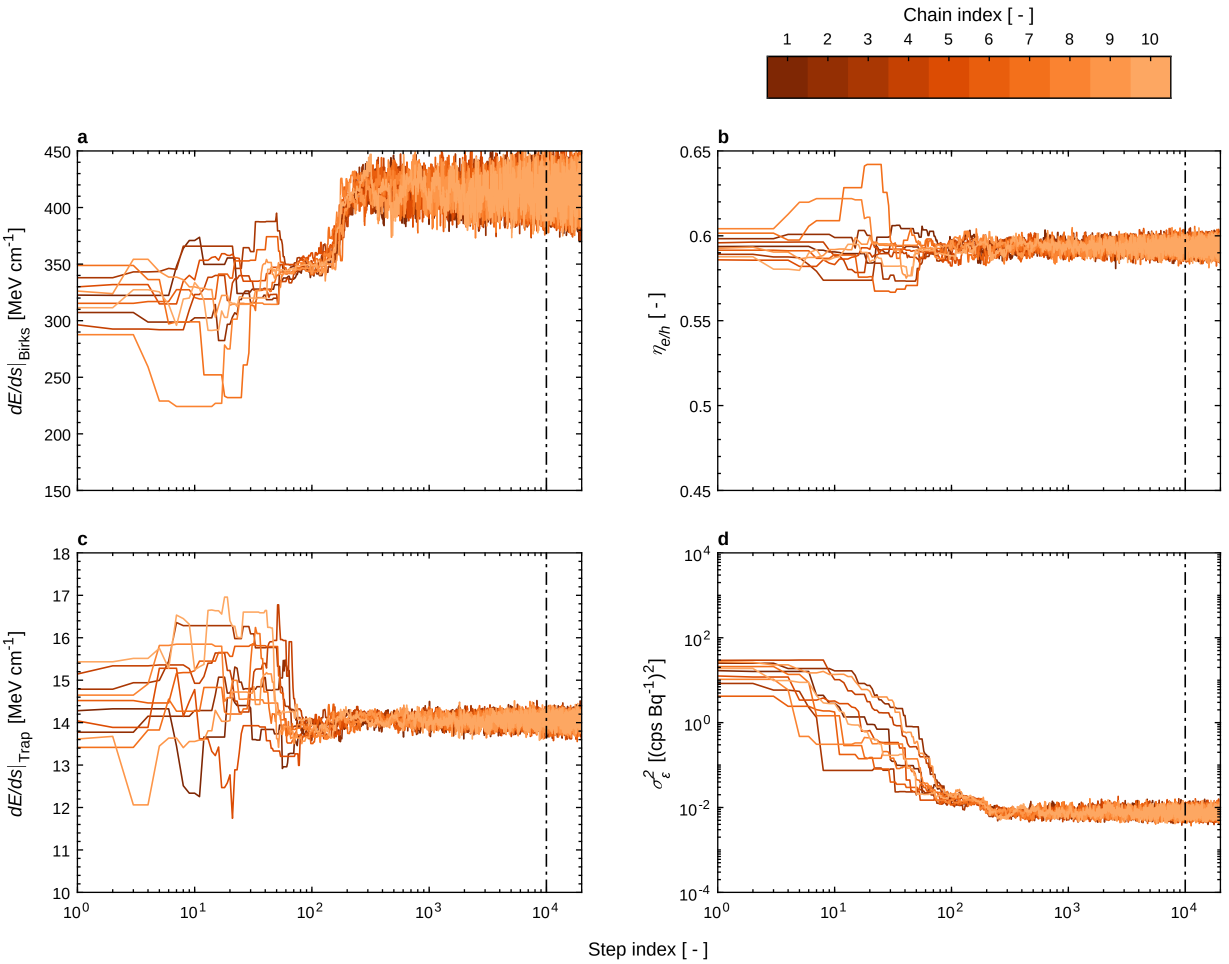

Fig. S3: **Markov Chain Monte Carlo trace plots for crystal 2.** These graphs show the sample values of the Markov Chain Monte Carlo algorithm [34] for each individual Markov chain and model parameter resulting from the single mode inversion pipeline applied to the scintillation crystal 2: **a** The Birks related stopping power parameter $dE/ds\mid_{\text{Birks}}$. **b** The free carrier fraction $\eta_{e/h}$. **c** The trapping related stopping power parameter $dE/ds\mid_{\text{Trap}}$. **d** The discrepancy model variance σ_ε^2. In addition, the burn-in threshold is highlighted as a dashed-dotted black line in each graph.

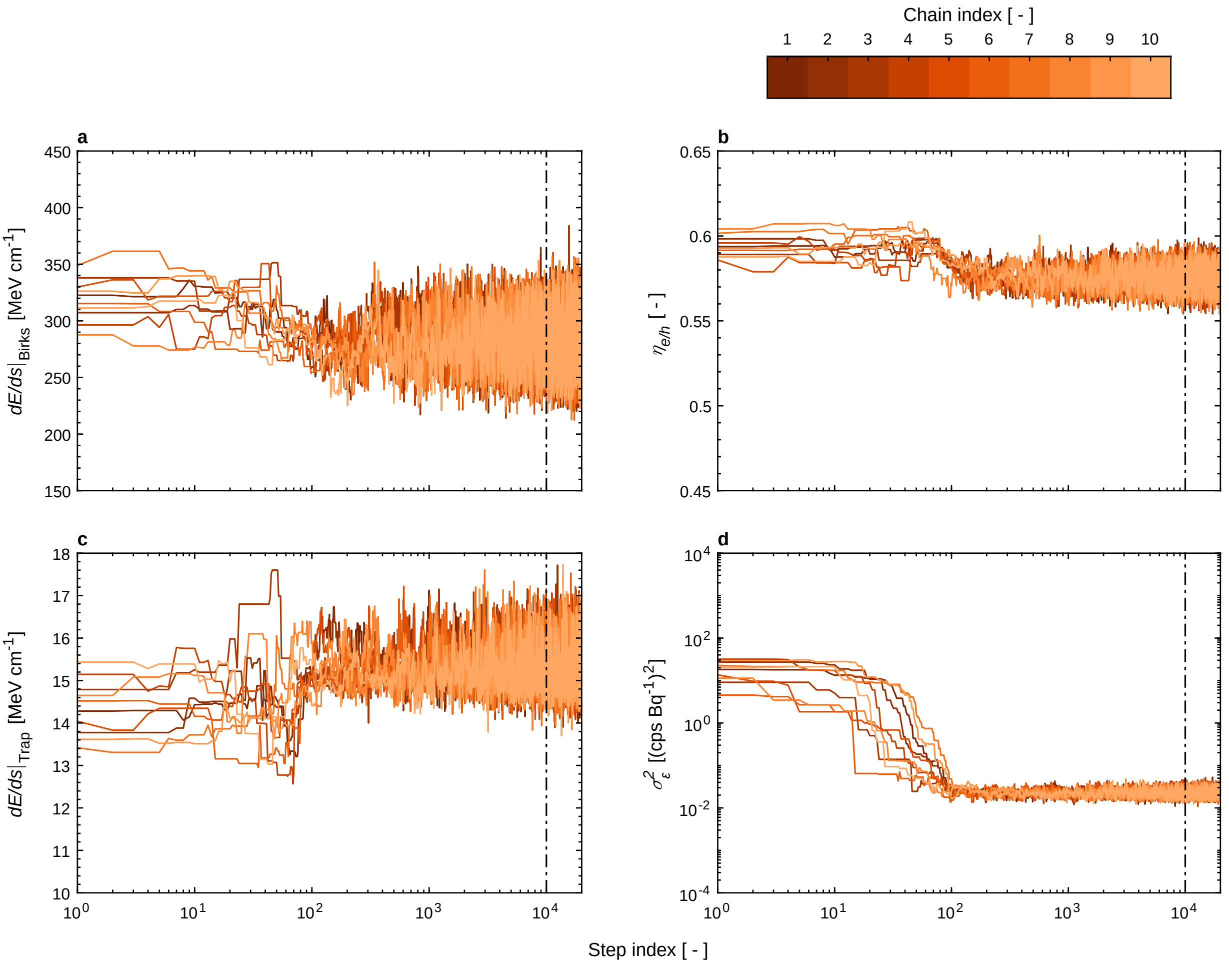

Fig. S4: **Markov Chain Monte Carlo trace plots for crystal 3.** These graphs show the sample values of the Markov Chain Monte Carlo algorithm [34] for each individual Markov chain and model parameter resulting from the single mode inversion pipeline applied to the scintillation crystal 3: **a** The Birks related stopping power parameter $dE/ds\ |_{\text{Birks}}$. **b** The free carrier fraction $\eta_{e/h}$. **c** The trapping related stopping power parameter $dE/ds\ |_{\text{Trap}}$. **d** The discrepancy model variance σ^2_ε. In addition, the burn-in threshold is highlighted as a dashed-dotted black line in each graph.

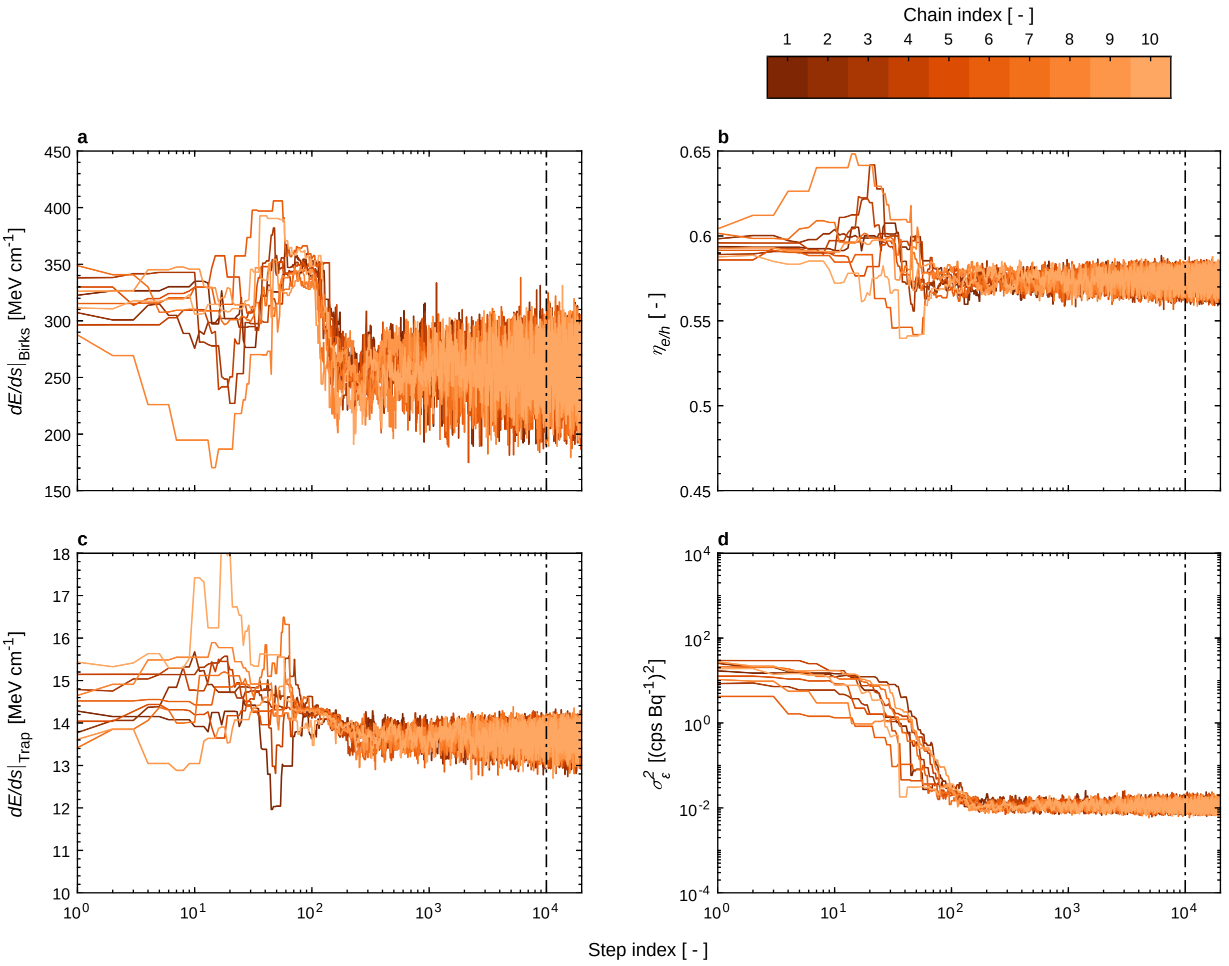

Fig. S5: **Markov Chain Monte Carlo trace plots for crystal 4.** These graphs show the sample values of the Markov Chain Monte Carlo algorithm [34] for each individual Markov chain and model parameter resulting from the single mode inversion pipeline applied to the scintillation crystal 4: **a** The Birks related stopping power parameter $dE/ds\mid_{\text{Birks}}$. **b** The free carrier fraction $\eta_{e/h}$. **c** The trapping related stopping power parameter $dE/ds\mid_{\text{Trap}}$. **d** The discrepancy model variance σ^2_ε. In addition, the burn-in threshold is highlighted as a dashed-dotted black line in each graph.

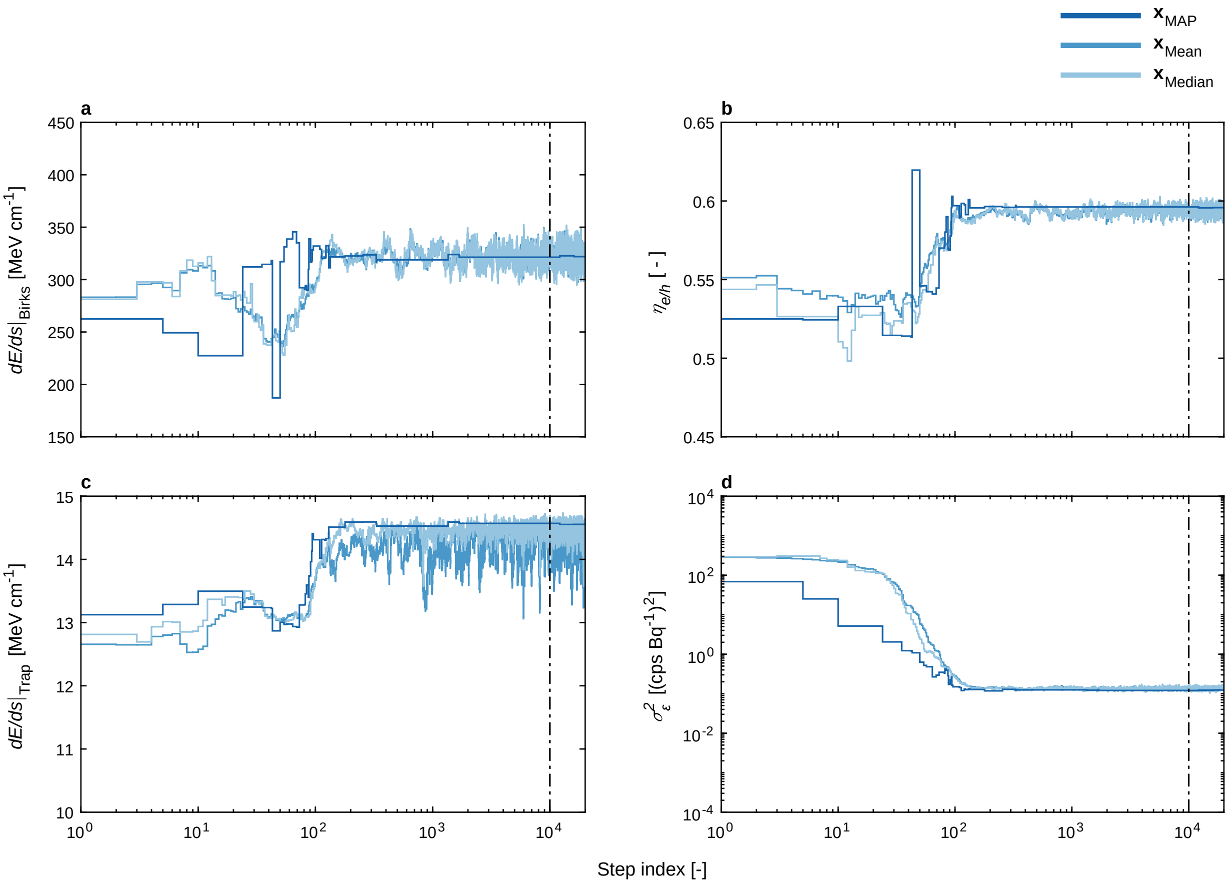

Fig. S6: **Posterior point estimator convergence for the sum mode.** These graphs show the convergence of the posterior point estimators, i.e. the maximum a posteriori (MAP) probability estimate $\mathbf{x}_{\text{MAP}}$, the posterior mean $\mathbf{x}_{\text{Mean}}$ and the posterior median $\mathbf{x}_{\text{Median}}$, as a function of the Markov Chain Monte Carlo steps and each individual model parameter resulting from the sum mode inversion pipeline applied to the sum channel: **a** The Birks related stopping power parameter $dE/ds\ |_{\text{Birks}}$. **b** The free carrier fraction $\eta_{e/h}$. **c** The trapping related stopping power parameter $dE/ds\ |_{\text{Trap}}$. **d** The discrepancy model variance σ_ε^2. In addition, the burn-in threshold is highlighted as a dashed-dotted black line in each graph.

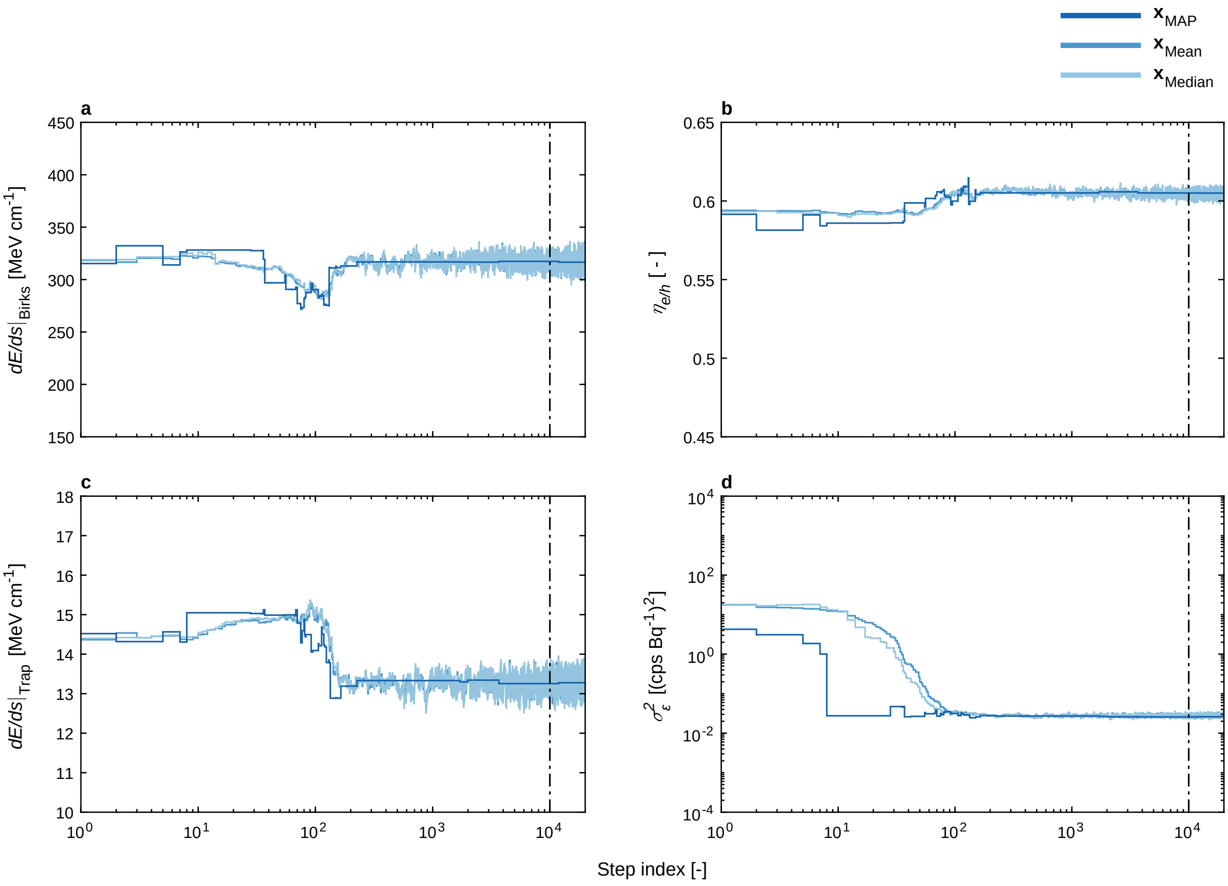

Fig. S7: **Posterior point estimator convergence for crystal 1.** These graphs show the convergence of the posterior point estimators, i.e. the maximum a posteriori (MAP) probability estimate $\mathbf{x}_{\mathrm{MAP}}$, the posterior mean $\mathbf{x}_{\mathrm{Mean}}$ and the posterior median $\mathbf{x}_{\mathrm{Median}}$, as a function of the Markov Chain Monte Carlo steps and each individual model parameter resulting from the single mode inversion pipeline applied to the scintillation crystal 1: **a** The Birks related stopping power parameter $dE/ds\,|_{\mathrm{Birks}}$. **b** The free carrier fraction $\eta_{e/h}$. **c** The trapping related stopping power parameter $dE/ds\,|_{\mathrm{Trap}}$. **d** The discrepancy model variance σ_ε^2. In addition, the burn-in threshold is highlighted as a dashed-dotted black line in each graph.

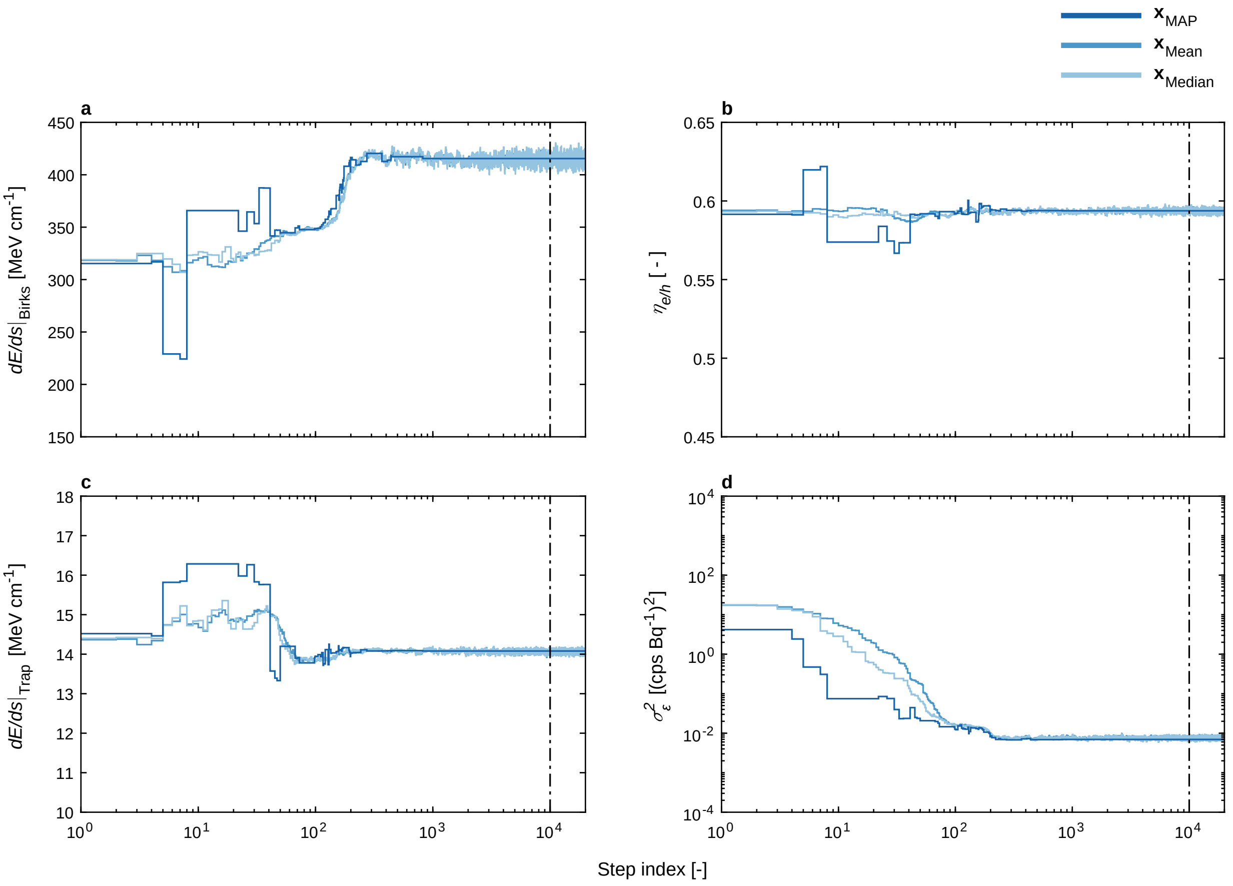

Fig. S8: **Posterior point estimator convergence for crystal 2.** These graphs show the convergence of the posterior point estimators, i.e. the maximum a posteriori (MAP) probability estimate $\mathbf{x}_{\text{MAP}}$, the posterior mean $\mathbf{x}_{\text{Mean}}$ and the posterior median $\mathbf{x}_{\text{Median}}$, as a function of the Markov Chain Monte Carlo steps and each individual model parameter resulting from the single mode inversion pipeline applied to the scintillation crystal 2: **a** The Birks related stopping power parameter $dE/ds\ |_{\text{Birks}}$. **b** The free carrier fraction $\eta_{e/h}$. **c** The trapping related stopping power parameter $dE/ds\ |_{\text{Trap}}$. **d** The discrepancy model variance σ_ε^2. In addition, the burn-in threshold is highlighted as a dashed-dotted black line in each graph.

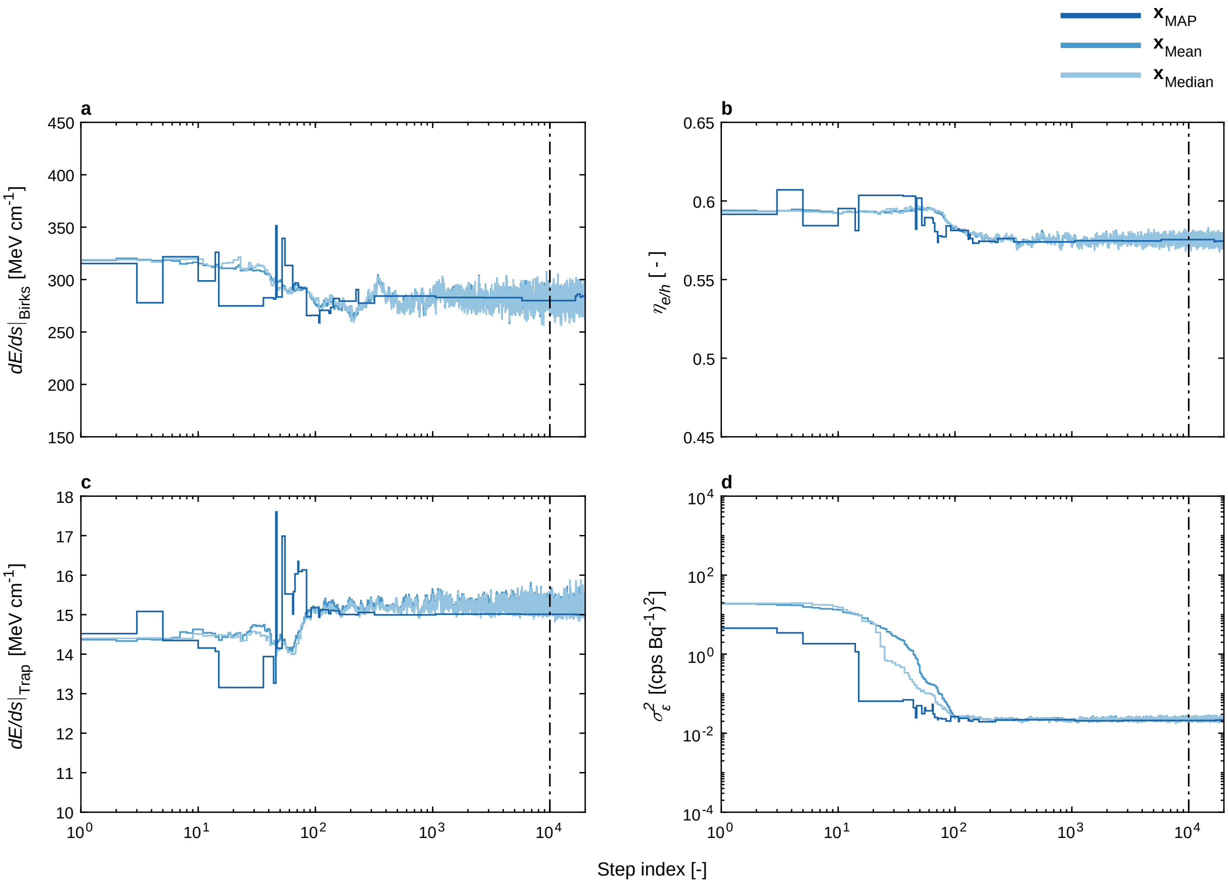

Fig. S9: **Posterior point estimator convergence for crystal 3.** These graphs show the convergence of the posterior point estimators, i.e. the maximum a posteriori (MAP) probability estimate $\mathbf{x}_{\mathrm{MAP}}$, the posterior mean $\mathbf{x}_{\mathrm{Mean}}$ and the posterior median $\mathbf{x}_{\mathrm{Median}}$, as a function of the Markov Chain Monte Carlo steps and each individual model parameter resulting from the single mode inversion pipeline applied to the scintillation crystal 3: **a** The Birks related stopping power parameter $dE/ds\,|_{\mathrm{Birks}}$. **b** The free carrier fraction $\eta_{e/h}$. **c** The trapping related stopping power parameter $dE/ds\,|_{\mathrm{Trap}}$. **d** The discrepancy model variance σ_{ε}^{2}. In addition, the burn-in threshold is highlighted as a dashed-dotted black line in each graph.

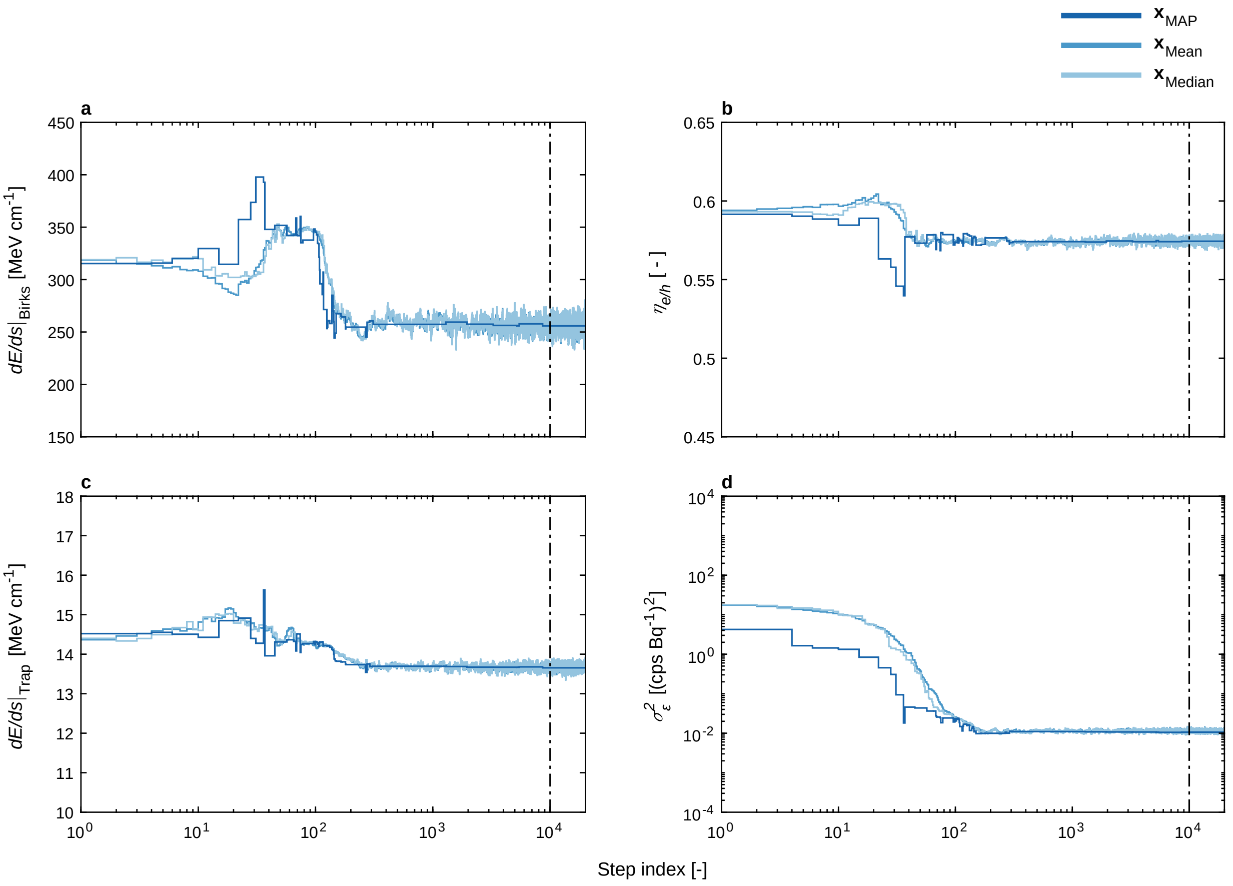

Fig. S10: **Posterior point estimator convergence for crystal 4.** These graphs show the convergence of the posterior point estimators, i.e. the maximum a posteriori (MAP) probability estimate $\mathbf{x}_{\mathrm{MAP}}$, the posterior mean $\mathbf{x}_{\mathrm{Mean}}$ and the posterior median $\mathbf{x}_{\mathrm{Median}}$, as a function of the Markov Chain Monte Carlo steps and each individual model parameter resulting from the single mode inversion pipeline applied to the scintillation crystal 4: **a** The Birks related stopping power parameter $dE/ds\mid_{\mathrm{Birks}}$. **b** The free carrier fraction $\eta_{e/h}$. **c** The trapping related stopping power parameter $dE/ds\mid_{\mathrm{Trap}}$. **d** The discrepancy model variance σ_ε^2. In addition, the burn-in threshold is highlighted as a dashed-dotted black line in each graph.

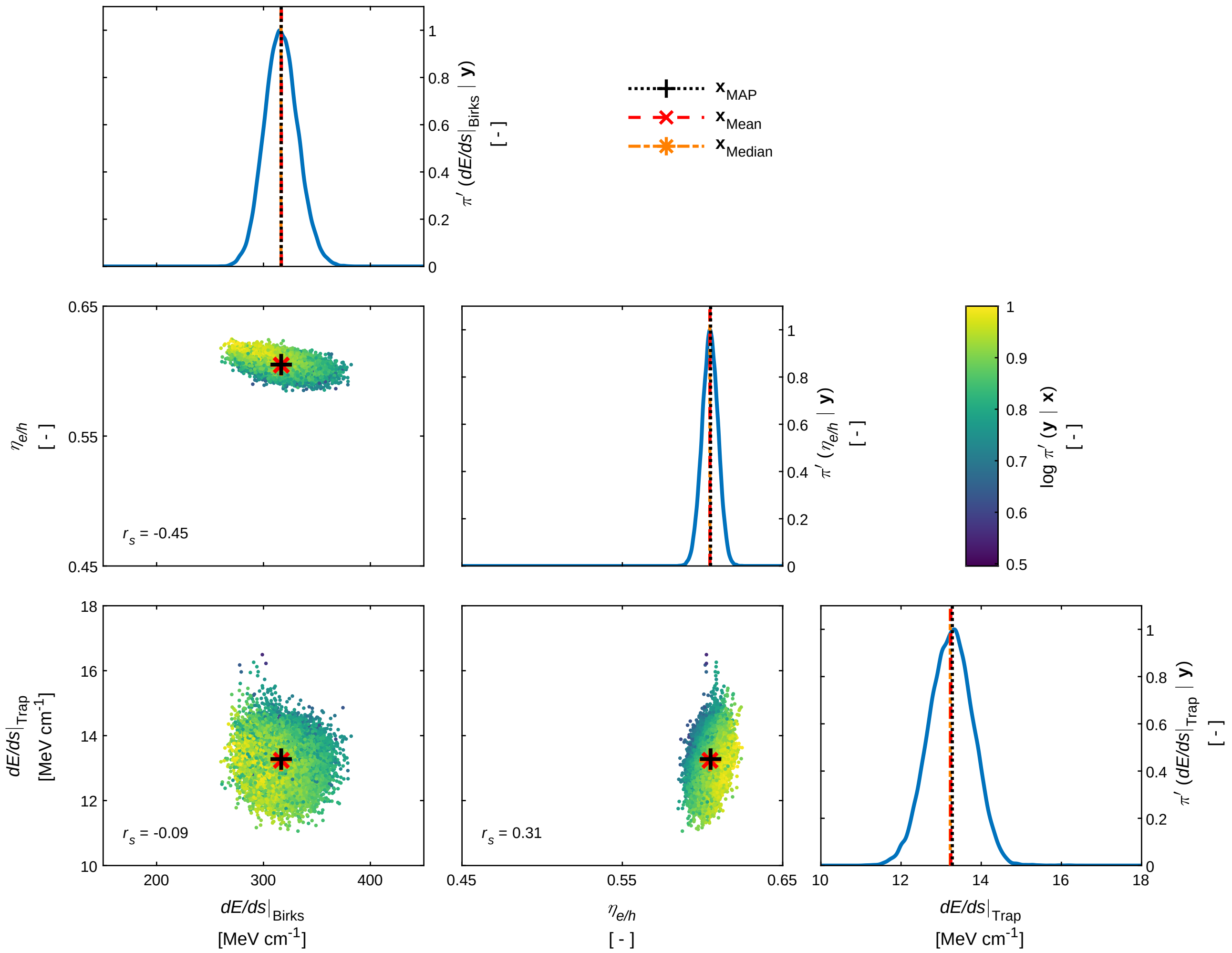

Fig. S11: **Posterior distribution estimate for crystal 1.** As a result of the single mode inversion pipeline applied to the scintillation crystal 1, the off-diagonal subfigures present samples from the multivariate posterior marginals given the experimental dataset $\mathbf{y}$ for the model parameters $\mathbf{x} := \left(dE/ds\,|_{\text{Birks}},\ \eta_{e/h},\ dE/ds\,|_{\text{Trap}}\right)^{\intercal}$. We colored these samples by the corresponding normalized multivariate log-likelihood function values $\log \pi' (\mathbf{y} \mid \mathbf{x})$. In addition, the Spearman's rank correlation coefficient r_s is provided for the model parameters in the corresponding off-diagonal subfigures. The subfigures on the diagonal axis highlight the normalized univariate marginal likelihood $\pi' (x \mid \mathbf{y})$ for the model parameter x. Both, the univariate and multivariate likelihood values, were normalized by their corresponding global maxima. Derived posterior point estimators, i.e. the maximum a posteriori (MAP) probability estimate $\mathbf{x}_{\text{MAP}}$, the posterior mean $\mathbf{x}_{\text{Mean}}$ and the posterior median $\mathbf{x}_{\text{Median}}$, are indicated as well in each subfigure.

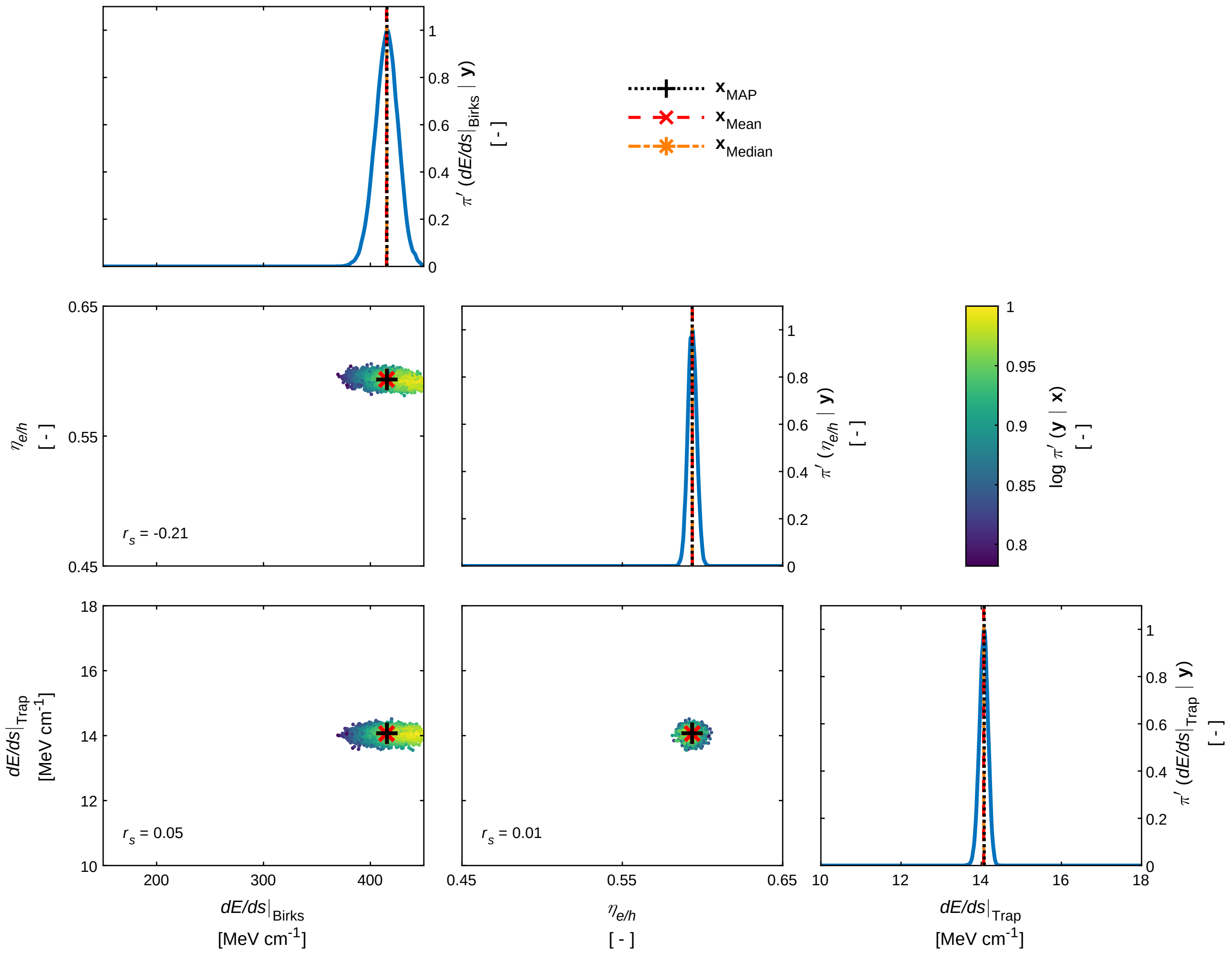

Fig. S12: **Posterior distribution estimate for crystal 2.** As a result of the single mode inversion pipeline applied to the scintillation crystal 2, the off-diagonal subfigures present samples from the multivariate posterior marginals given the experimental dataset $\mathbf{y}$ for the model parameters $\mathbf{x} := \left(dE/ds\,|_{\text{Birks}},\ \eta_{e/h},\ dE/ds\,|_{\text{Trap}}\right)^{\mathsf{T}}$. We colored these samples by the corresponding normalized multivariate log-likelihood function values $\log \pi'(\mathbf{y} \mid \mathbf{x})$. In addition, the Spearman's rank correlation coefficient r_s is provided for the model parameters in the corresponding off-diagonal subfigures. The subfigures on the diagonal axis highlight the normalized univariate marginal likelihood $\pi'(x \mid \mathbf{y})$ for the model parameter x. Both, the univariate and multivariate likelihood values, were normalized by their corresponding global maxima. Derived posterior point estimators, i.e. the maximum a posteriori (MAP) probability estimate $\mathbf{x}_{\text{MAP}}$, the posterior mean $\mathbf{x}_{\text{Mean}}$ and the posterior median $\mathbf{x}_{\text{Median}}$, are indicated as well in each subfigure.

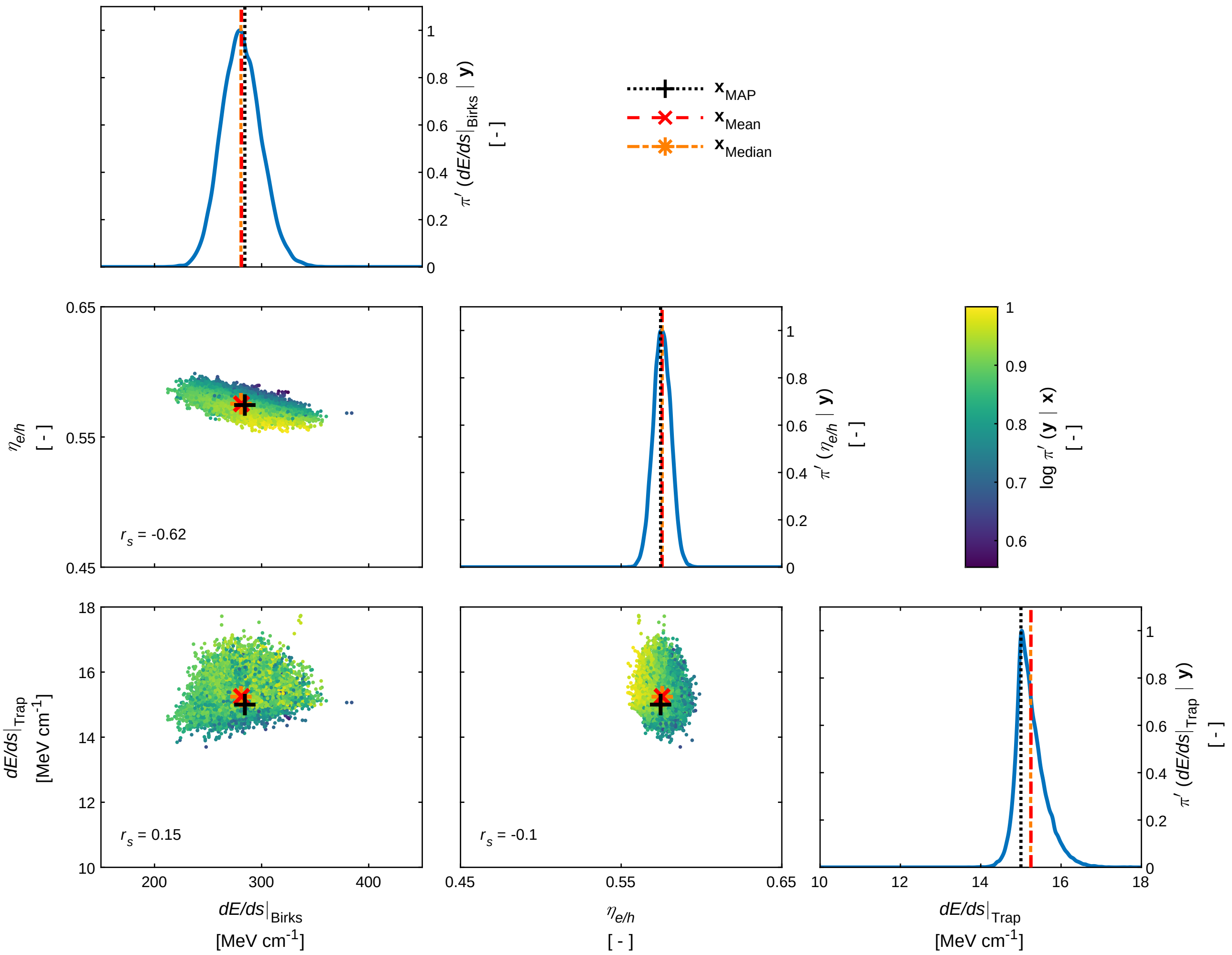

Fig. S13: **Posterior distribution estimate for crystal 3.** As a result of the single mode inversion pipeline applied to the scintillation crystal 3, the off-diagonal subfigures present samples from the multivariate posterior marginals given the experimental dataset $\mathbf{y}$ for the model parameters $\mathbf{x} := \left(dE/ds\,|_{\text{Birks}},\ \eta_{e/h},\ dE/ds\,|_{\text{Trap}}\right)^{\mathsf{T}}$. We colored these samples by the corresponding normalized multivariate log-likelihood function values $\log \pi' \left(\mathbf{y} \mid \mathbf{x}\right)$. In addition, the Spearman's rank correlation coefficient r_s is provided for the model parameters in the corresponding off-diagonal subfigures. The subfigures on the diagonal axis highlight the normalized univariate marginal likelihood $\pi' \left(x \mid \mathbf{y}\right)$ for the model parameter x. Both, the univariate and multivariate likelihood values, were normalized by their corresponding global maxima. Derived posterior point estimators, i.e. the maximum a posteriori (MAP) probability estimate $\mathbf{x}_{\text{MAP}}$, the posterior mean $\mathbf{x}_{\text{Mean}}$ and the posterior median $\mathbf{x}_{\text{Median}}$, are indicated as well in each subfigure.

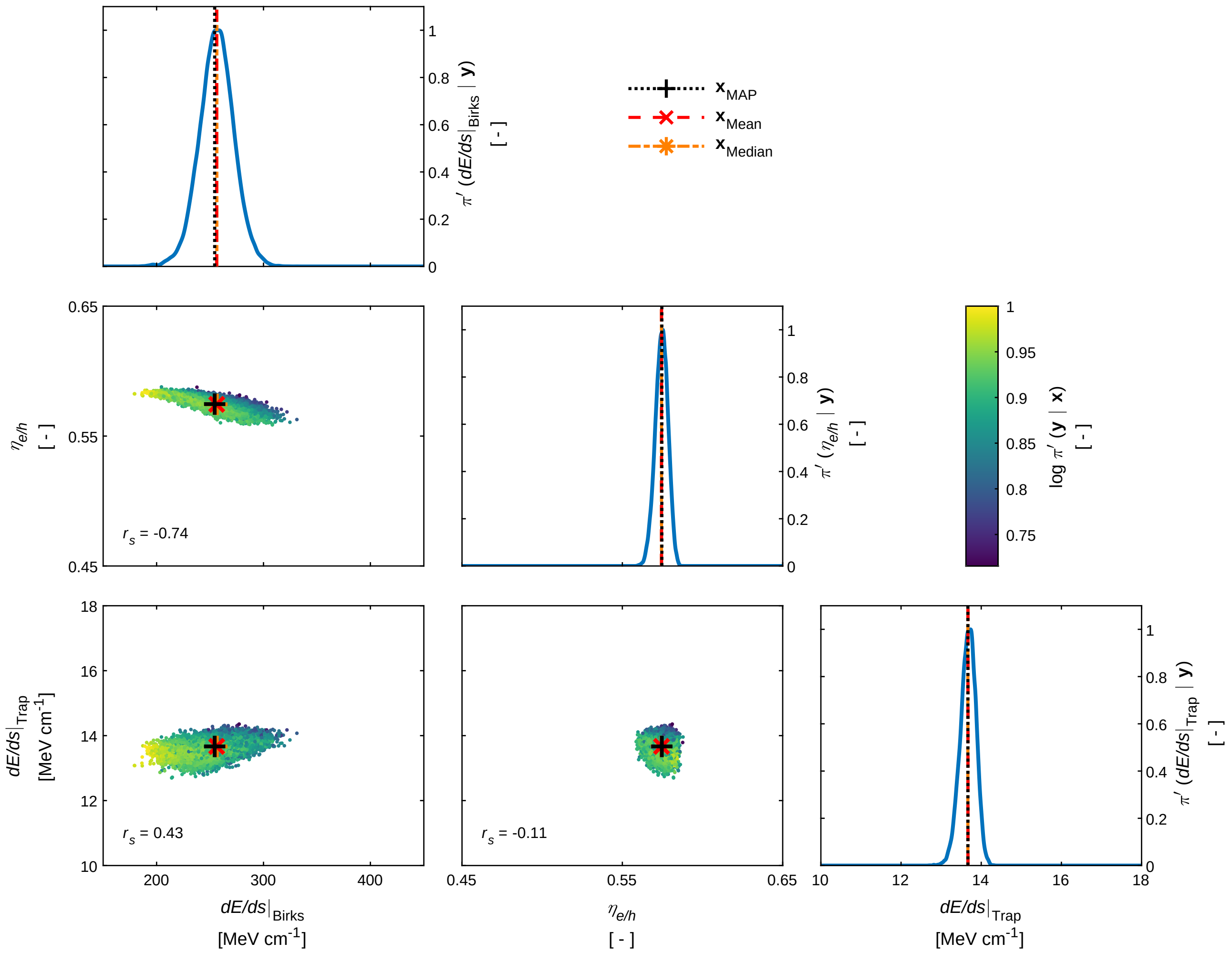

Fig. S14: **Posterior distribution estimate for crystal 4.** As a result of the single mode inversion pipeline applied to the scintillation crystal 4, the off-diagonal subfigures present samples from the multivariate posterior marginals given the experimental dataset $\mathbf{y}$ for the model parameters $\mathbf{x} := \left(dE/ds\mid_{\text{Birks}},\ \eta_{e/h},\ dE/ds\mid_{\text{Trap}}\right)^{\mathsf{T}}$. We colored these samples by the corresponding normalized multivariate log-likelihood function values $\log \pi'(\mathbf{y} \mid \mathbf{x})$. In addition, the Spearman's rank correlation coefficient r_s is provided for the model parameters in the corresponding off-diagonal subfigures. The subfigures on the diagonal axis highlight the normalized univariate marginal likelihood $\pi'(x \mid \mathbf{y})$ for the model parameter x. Both, the univariate and multivariate likelihood values, were normalized by their corresponding global maxima. Derived posterior point estimators, i.e. the maximum a posteriori (MAP) probability estimate $\mathbf{x}_{\text{MAP}}$, the posterior mean $\mathbf{x}_{\text{Mean}}$ and the posterior median $\mathbf{x}_{\text{Median}}$, are indicated as well in each subfigure.

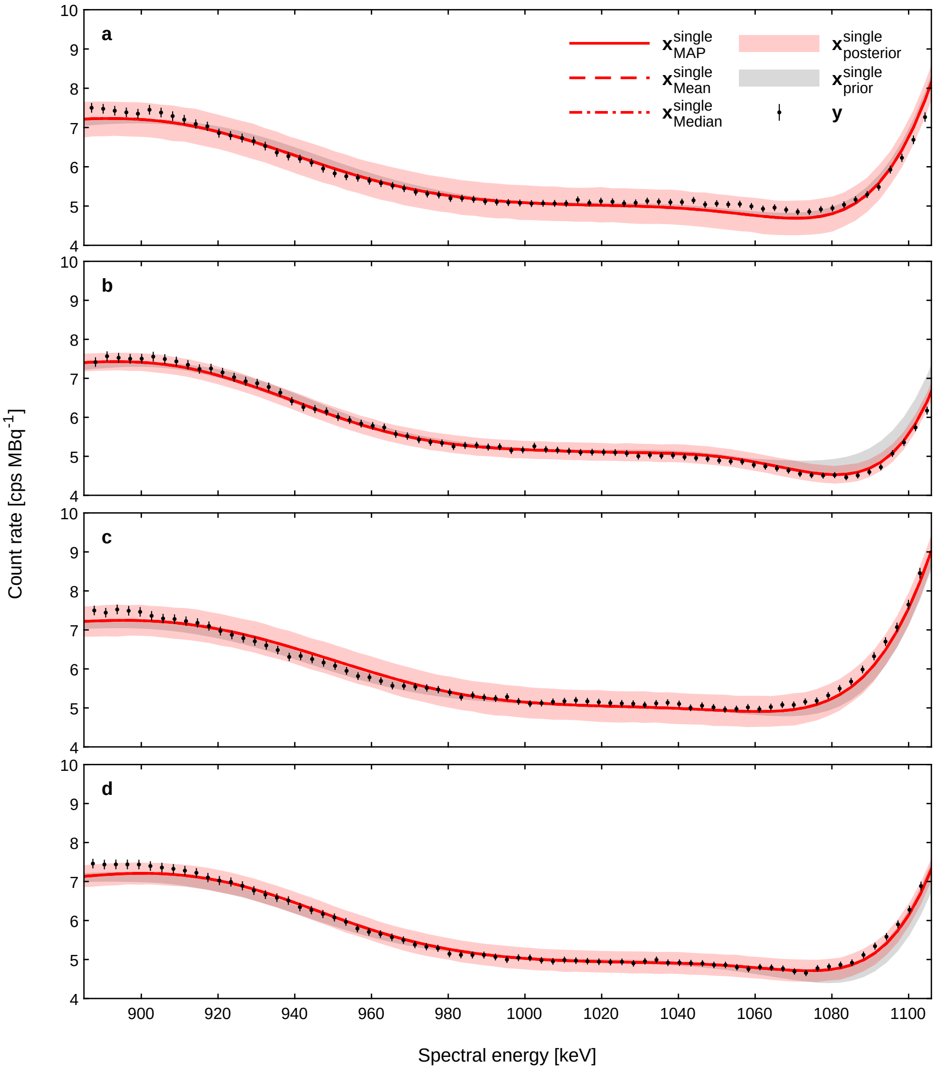

Fig. S15: **Compton edge predictions for the individual crystals.** Here, we show the prior and posterior predictive distributions using the 99% central credible interval for the individual scintillation crystals 1–4 (**a–d**) obtained by the single mode inversion pipeline applied to the corresponding crystals and the spectral Compton edge domain $\mathcal{D}_E := \{E : E_{\text{CE}} - 3 \cdot \sigma_{\text{tot}}\left(E_{\text{CE}}\right) \leq E \leq E_{\text{FEP}} - 2 \cdot \sigma_{\text{tot}}\left(E_{\text{FEP}}\right)\}$ (cf. Methods in the main study). In addition, the experimental data $\mathbf{y}$ together with the posterior predictions using point estimators, i.e. the maximum a posteriori (MAP) probability estimate $\mathbf{x}_{\text{MAP}}^{\text{single}}$, the posterior mean $\mathbf{x}_{\text{Mean}}^{\text{single}}$ and the posterior median $\mathbf{x}_{\text{Median}}^{\text{single}}$, are indicated in each subfigure. Experimental uncertainties are provided as 1 standard deviation (SD) values (coverage factor $k = 1$).

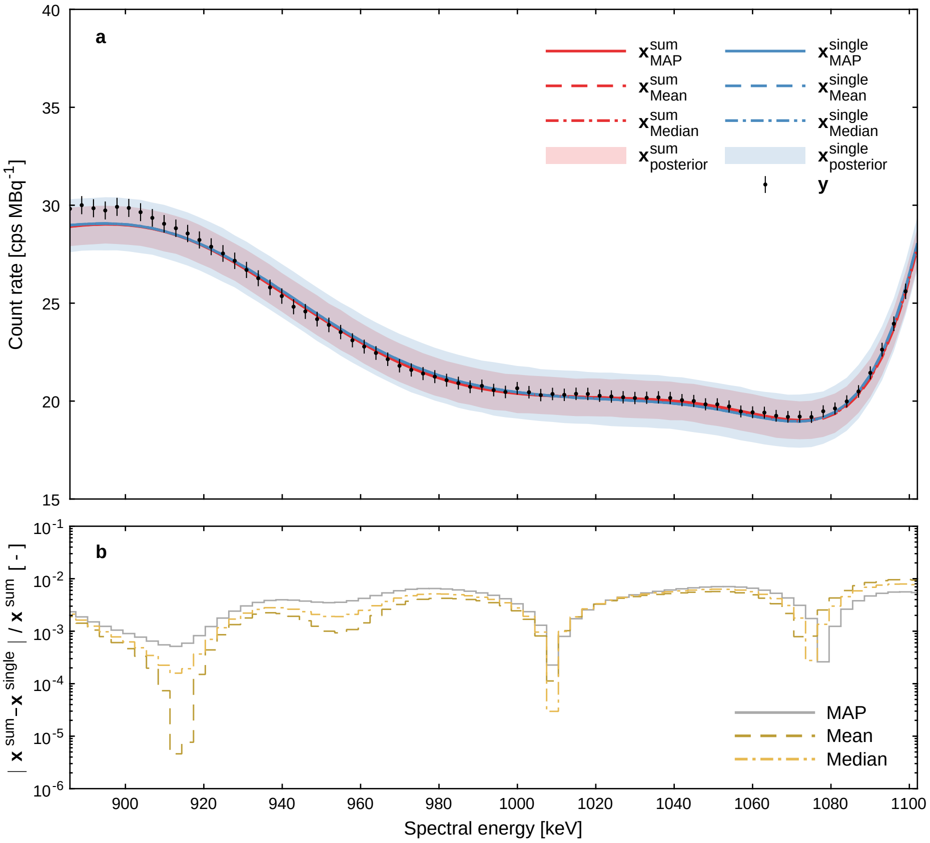

Fig. S16: **Spectral comparison of the sum and single mode inversion pipelines.** Here, we quantitatively compare the two inversion pipelines, sum and single, for the spectral Compton edge domain $\mathcal{D}_E := \{E : E_{\mathrm{CE}} - 3 \cdot \sigma_{\mathrm{tot}}\,(E_{\mathrm{CE}}) \leq E \leq E_{\mathrm{FEP}} - 2 \cdot \sigma_{\mathrm{tot}}\,(E_{\mathrm{FEP}})\}$ (cf. Methods in the main study). For that purpose, the posterior predictions for the individual scintillation crystals obtained by the single mode inversion pipeline are summed together and compared to the predictions for the sum channel obtained by the sum mode inversion pipeline. **a** In this graph, we show the prior and posterior predictive distributions using the 99% central credible interval for the two inversion pipelines, sum and single. In addition, the experimental data $\mathbf{y}$ together with the derived posterior predictions using point estimators, i.e. the maximum a posteriori (MAP) probability estimate $\mathbf{x}_{\mathrm{MAP}}$, the posterior mean $\mathbf{x}_{\mathrm{Mean}}$ and the posterior median $\mathbf{x}_{\mathrm{Median}}$, are indicated for each pipeline. Experimental uncertainties are provided as 1 standard deviation (SD) values (coverage factor $k = 1$). **b** In this subfigure, we present the relative difference between the two inversion pipelines, sum and single, for the posterior predictions shown in subfigure a, i.e. predictions using the three point estimators $\mathbf{x}_{\mathrm{MAP}}$, $\mathbf{x}_{\mathrm{Mean}}$ and $\mathbf{x}_{\mathrm{Median}}$.

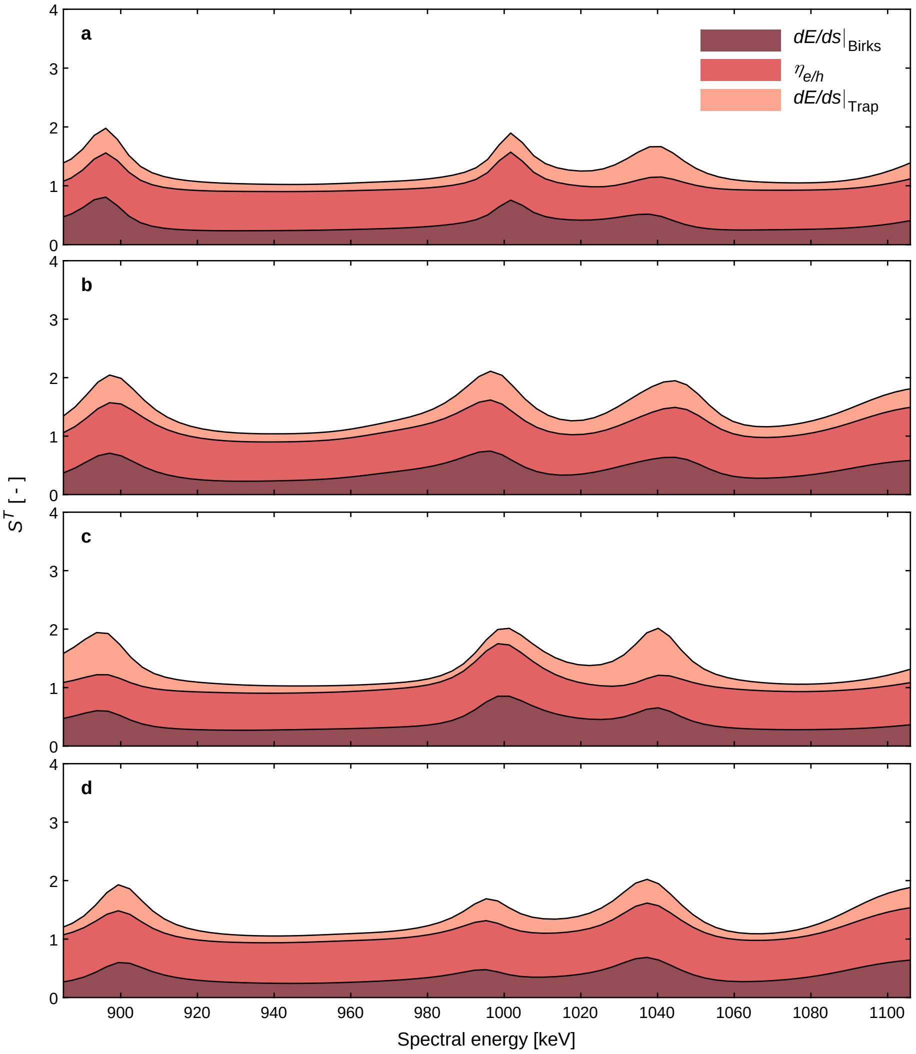

Fig. S17: **Hoeffding-Sobol decomposition for the individual crystals.** In this graph, we present the total Sobol indices S^T computed by the polynomial chaos expansion emulators [6] for the individual scintillation crystals 1–4 (**a–d**) following the single mode inversion pipeline. The total Sobol indices are computed for the individual non-proportional scintillation model parameters, i.e. the Birks related stopping power parameter $dE/ds\mid_{\text{Birks}}$, the free carrier fraction $\eta_{e/h}$ as well as the trapping related stopping power parameter $dE/ds\mid_{\text{Trap}}$, on the spectral Compton edge domain $\mathcal{D}_E \coloneqq \{E : E_{\text{CE}} - 3 \cdot \sigma_{\text{tot}}\left(E_{\text{CE}}\right) \leq E \leq E_{\text{FEP}} - 2 \cdot \sigma_{\text{tot}}\left(E_{\text{FEP}}\right)\}$ (cf. Methods in the main study).

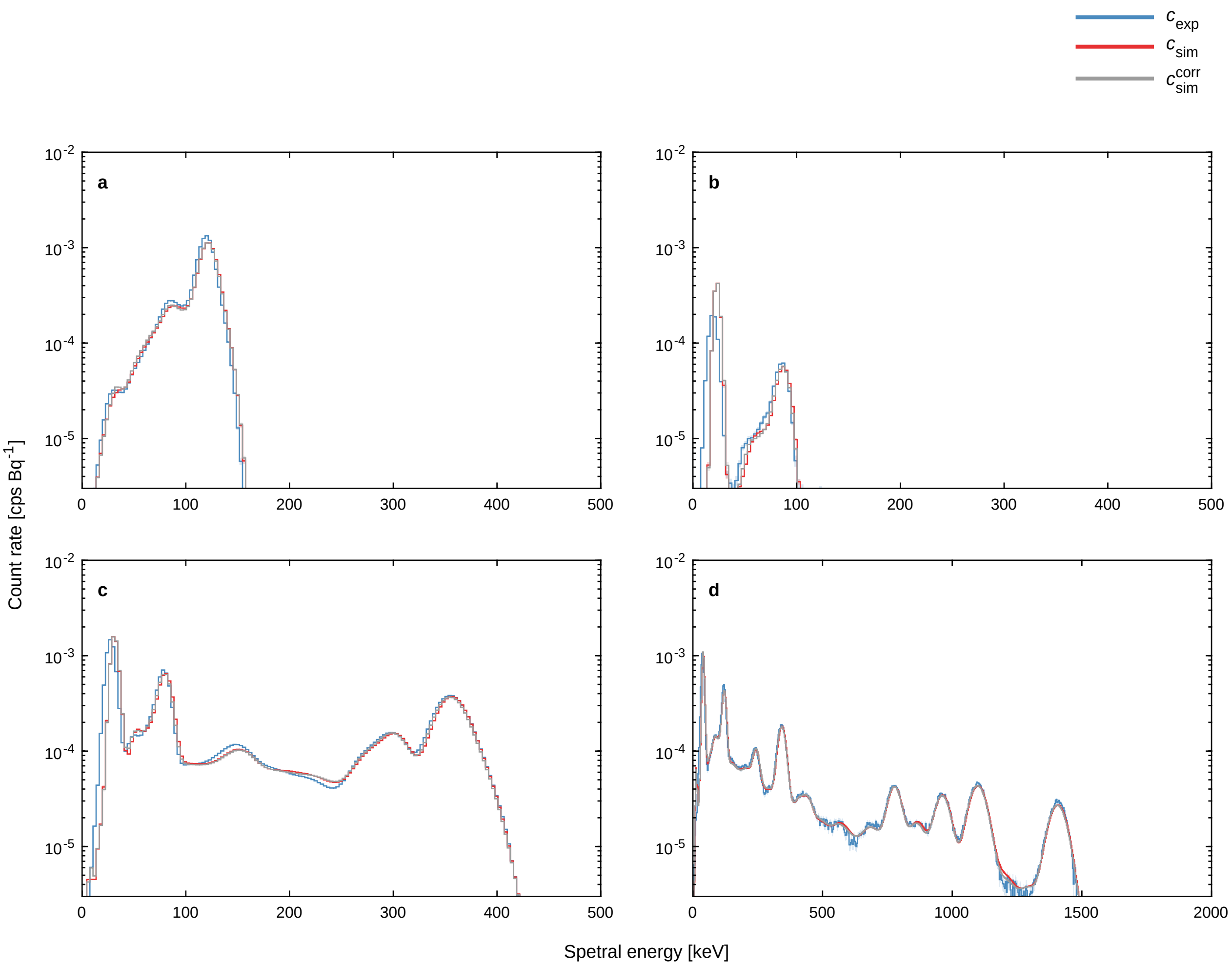

Fig. S18: Spectral detector response for ^{57}Co, ^{109}Cd, ^{133}Ba and ^{152}Eu. The measured and simulated spectral detector responses are shown for the sum channel using the four different calibrated radionuclide sources: **a** ^{57}Co, ^{109}Cd ($A = 1.113(18) \times 10^5$ Bq). **b** ^{109}Cd ($A = 7.38(15) \times 10^4$ Bq). **c** ^{133}Ba ($A = 2.152(32) \times 10^5$ Bq). **d** ^{152}Eu ($A = 1.973(30) \times 10^4$ Bq). The measured net count rate c_{exp} as well as the simulated net count rate adopting a proportional scintillation model c_{sim} were presented already elsewhere [9]. We obtained the simulated net count rate $c_{\mathrm{sim}}^{\mathrm{corr}}$ the same way as c_{sim} but accounted for the non-proportional scintillation effects by the sum mode inversion pipeline presented in this study. For the calibration, we used the ^{60}Co dataset [9]. For all graphs presented in this figure, uncertainties are provided as 1 standard deviation (SD) shaded areas (coverage factor $k = 1$). These uncertainties are only visible for c_{exp}.

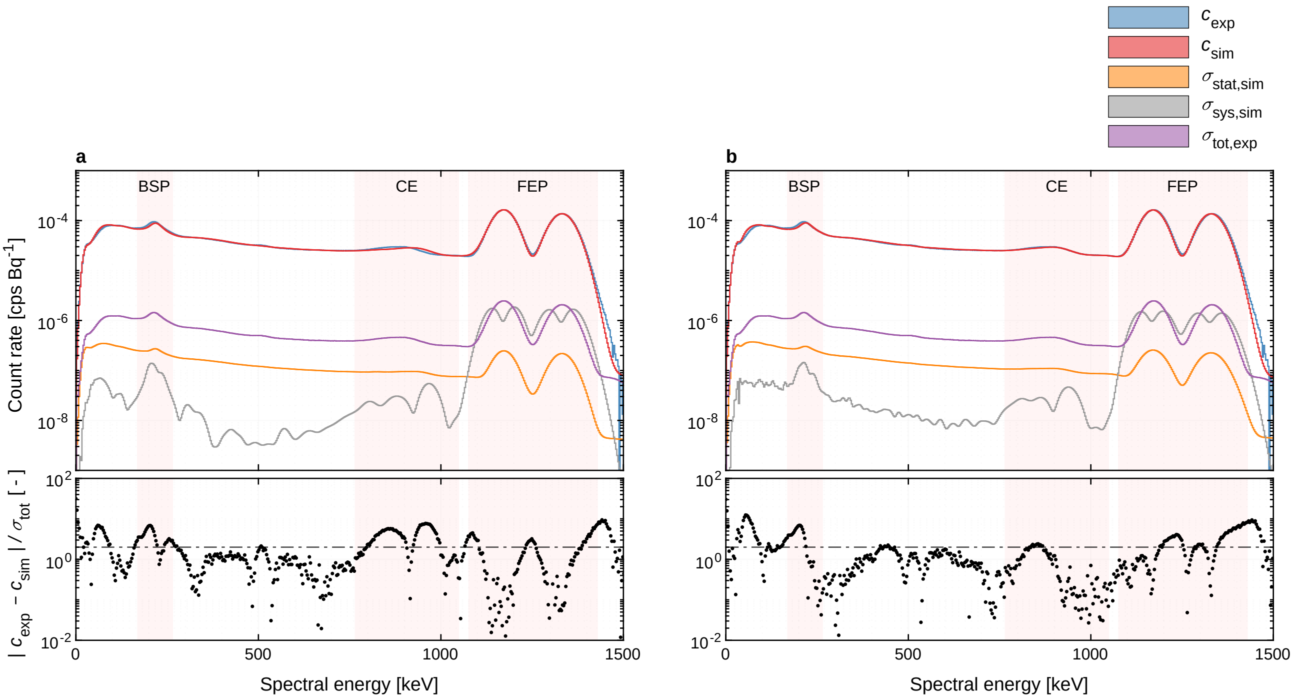

Fig. S19: **Uncertainty quantification for the ^{60}Co spectral detector response.** The measured and simulated mean net count rates c_{exp} and c_{sim} are shown for the sum channel using a ^{60}Co calibrated radionuclide source ($A = 3.08(5) \times 10^5$ Bq) together with the corresponding uncertainty estimates, i.e. the combined statistical and systematic measured uncertainty $\sigma_{\text{tot,exp}}$, the simulated statistical uncertainty $\sigma_{\text{stat,sim}}$ as well as the simulated systematic uncertainty $\sigma_{\text{sys,sim}}$, using 1 standard deviation values. The measurement results were presented already elsewhere [9]. Two different scintillation models have been used for the simulations: **a** Proportional scintillation model published in [9]. **b** Bayesian calibrated non-proportional scintillation model obtained by the sum mode inversion pipeline presented in this study. Distinct spectral regions, i.e. the backscatter peak (BSP), the Compton edge (CE) as well as the full energy peaks (FEP) are highlighted for both graphs. Note that the highlighted CE region refers to the lower Compton edge at 963.419(3) keV associated with the photon emission line at 1173.228(3) keV. The normalized residual level $\mid c_{\text{exp}} - c_{\text{sim}} \mid / \sigma_{\text{tot}}$ with $\sigma_{\text{tot}} := \sqrt{\sigma^2_{\text{tot,exp}} + \sigma^2_{\text{tot,sim}}}$ for a coverage factor of 2 is marked with the horizontal dash-dotted black line in the lower subfigures. More information on the numerical computation of the uncertainty estimates can be found in Section S1.3 and [9].

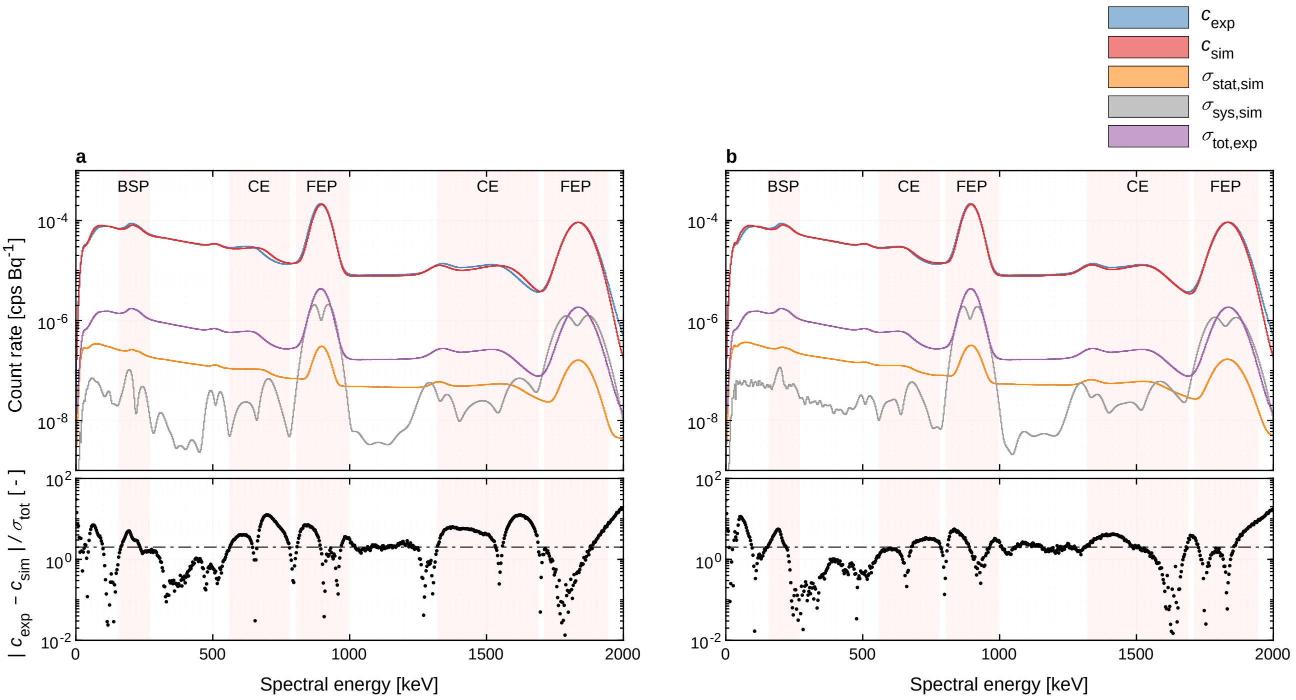

Fig. S20: **Uncertainty quantification for the ^{88}Y spectral detector response.** The measured and simulated mean net count rates c_{exp} and c_{sim} are shown for the sum channel using a ^{88}Y calibrated radionuclide source ($A = 6.83(14) \times 10^5$ Bq) together with the corresponding uncertainty estimates, i.e. the combined statistical and systematic measured uncertainty $\sigma_{\mathrm{tot,exp}}$, the simulated statistical uncertainty $\sigma_{\mathrm{stat,sim}}$ as well as the simulated systematic uncertainty $\sigma_{\mathrm{sys,sim}}$, using 1 standard deviation values. The measurement results were presented already elsewhere [9]. Two different scintillation models have been used for the simulations: **a** Proportional scintillation model published in [9]. **b** Bayesian calibrated non-proportional scintillation model obtained by the sum mode inversion pipeline presented in this study. Distinct spectral regions, i.e. the backscatter peak (BSP), the Compton edges (CE) as well as the full energy peaks (FEP) are highlighted for both graphs. The normalized residual level $\mid c_{\mathrm{exp}} - c_{\mathrm{sim}} \mid / \sigma_{\mathrm{tot}}$ with $\sigma_{\mathrm{tot}} := \sqrt{\sigma_{\mathrm{tot,exp}}^2 + \sigma_{\mathrm{tot,sim}}^2}$ for a coverage factor of 2 is marked with the horizontal dash-dotted black line in the lower subfigures. More information on the numerical computation of the uncertainty estimates can be found in Section S1.3 and [9].

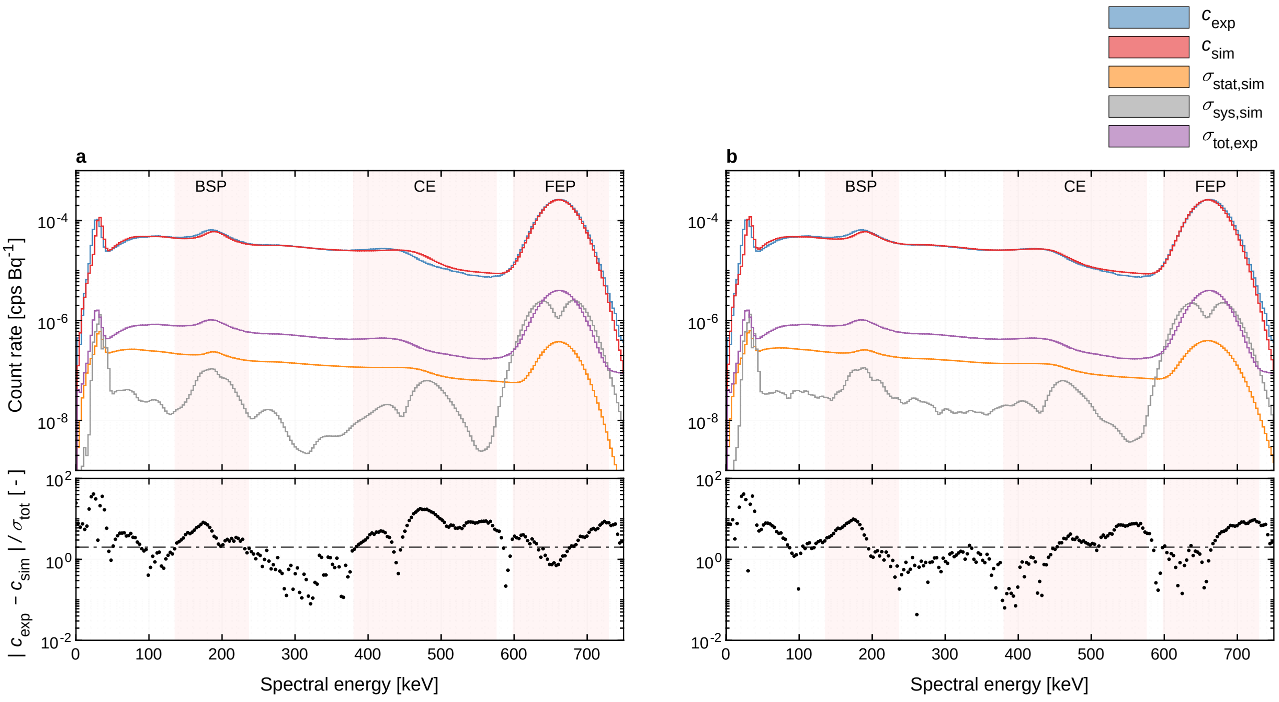

Fig. S21: **Uncertainty quantification for the ^{137}Cs spectral detector response.** The measured and simulated mean net count rates c_{exp} and c_{sim} are shown for the sum channel using a ^{137}Cs calibrated radionuclide source ($A = 2.266(34) \times 10^5$ Bq) together with the corresponding uncertainty estimates, i.e. the combined statistical and systematic measured uncertainty $\sigma_{\mathrm{tot,exp}}$, the simulated statistical uncertainty $\sigma_{\mathrm{stat,sim}}$ as well as the simulated systematic uncertainty $\sigma_{\mathrm{sys,sim}}$, using 1 standard deviation values. The measurement results were presented already elsewhere [9]. Two different scintillation models have been used for the simulations: **a** Proportional scintillation model published in [9]. **b** Bayesian calibrated non-proportional scintillation model obtained by the sum mode inversion pipeline presented in this study. Distinct spectral regions, i.e. the backscatter peak (BSP), the Compton edge (CE) as well as the full energy peak (FEP) are highlighted for both graphs. The normalized residual level $\mid c_{\mathrm{exp}} - c_{\mathrm{sim}} \mid / \sigma_{\mathrm{tot}}$ with $\sigma_{\mathrm{tot}} := \sqrt{\sigma^2_{\mathrm{tot,exp}} + \sigma^2_{\mathrm{tot,sim}}}$ for a coverage factor of 2 is marked with the horizontal dash-dotted black line in the lower subfigures. More information on the numerical computation of the uncertainty estimates can be found in Section S1.3 and [9].

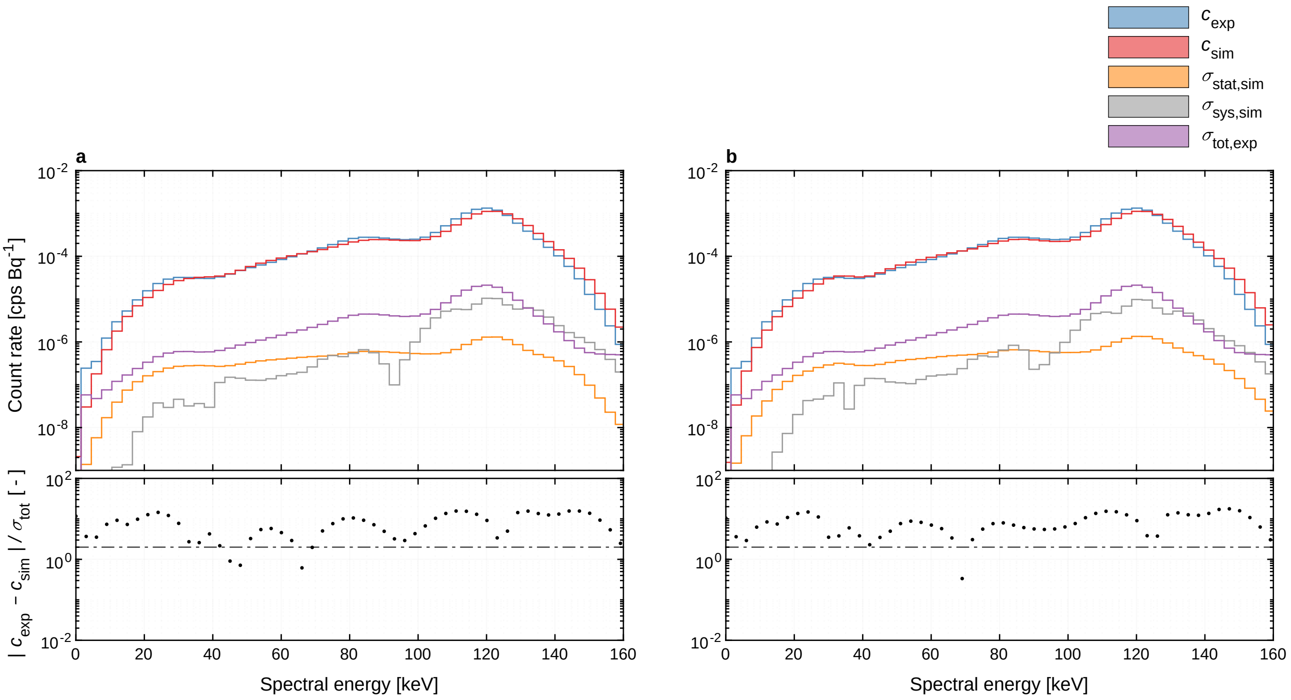

Fig. S22: **Uncertainty quantification for the ^{57}Co spectral detector response.** The measured and simulated mean net count rates c_{exp} and c_{sim} are shown for the sum channel using a ^{57}Co calibrated radionuclide source ($A = 1.113(18) \times 10^5$ Bq) together with the corresponding uncertainty estimates, i.e. the combined statistical and systematic measured uncertainty $\sigma_{\text{tot,exp}}$, the simulated statistical uncertainty $\sigma_{\text{stat,sim}}$ as well as the simulated systematic uncertainty $\sigma_{\text{sys,sim}}$, using 1 standard deviation values. The measurement results were presented already elsewhere [9]. Two different scintillation models have been used for the simulations: **a** Proportional scintillation model published in [9]. **b** Bayesian calibrated non-proportional scintillation model obtained by the sum mode inversion pipeline presented in this study. The normalized residual level $\mid c_{\text{exp}} - c_{\text{sim}} \mid / \sigma_{\text{tot}}$ with $\sigma_{\text{tot}} := \sqrt{\sigma^2_{\text{tot,exp}} + \sigma^2_{\text{tot,sim}}}$ for a coverage factor of 2 is marked with the horizontal dash-dotted black line in the lower subfigures. More information on the numerical computation of the uncertainty estimates can be found in Section S1.3 and [9].

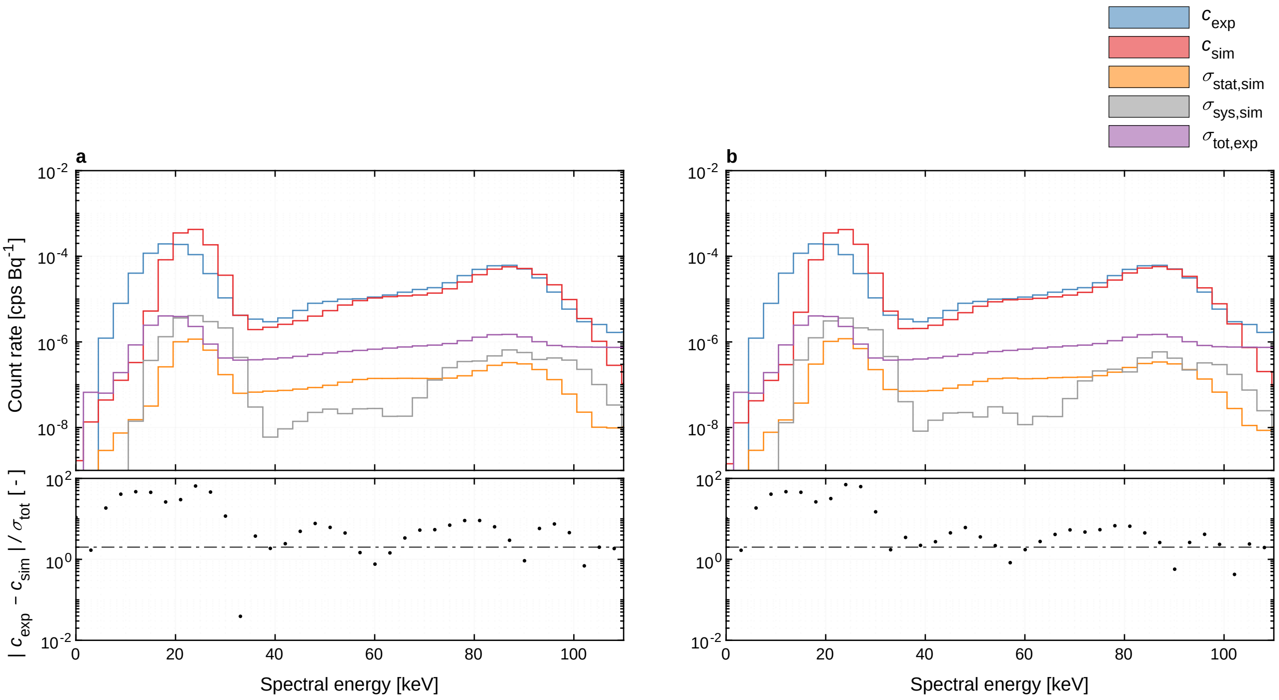

Fig. S23: **Uncertainty quantification for the ^{109}Cd spectral detector response.** The measured and simulated mean net count rates c_{exp} and c_{sim} are shown for the sum channel using a ^{109}Cd calibrated radionuclide source ($A = 7.38(15) \times 10^4$ Bq) together with the corresponding uncertainty estimates, i.e. the combined statistical and systematic measured uncertainty $\sigma_{\mathrm{tot,exp}}$, the simulated statistical uncertainty $\sigma_{\mathrm{stat,sim}}$ as well as the simulated systematic uncertainty $\sigma_{\mathrm{sys,sim}}$, using 1 standard deviation values. The measurement results were presented already elsewhere [9]. Two different scintillation models have been used for the simulations: **a** Proportional scintillation model published in [9]. **b** Bayesian calibrated non-proportional scintillation model obtained by the sum mode inversion pipeline presented in this study. The normalized residual level $\mid c_{\mathrm{exp}} - c_{\mathrm{sim}} \mid / \sigma_{\mathrm{tot}}$ with $\sigma_{\mathrm{tot}} := \sqrt{\sigma^2_{\mathrm{tot,exp}} + \sigma^2_{\mathrm{tot,sim}}}$ for a coverage factor of 2 is marked with the horizontal dash-dotted black line in the lower subfigures. More information on the numerical computation of the uncertainty estimates can be found in Section S1.3 and [9].

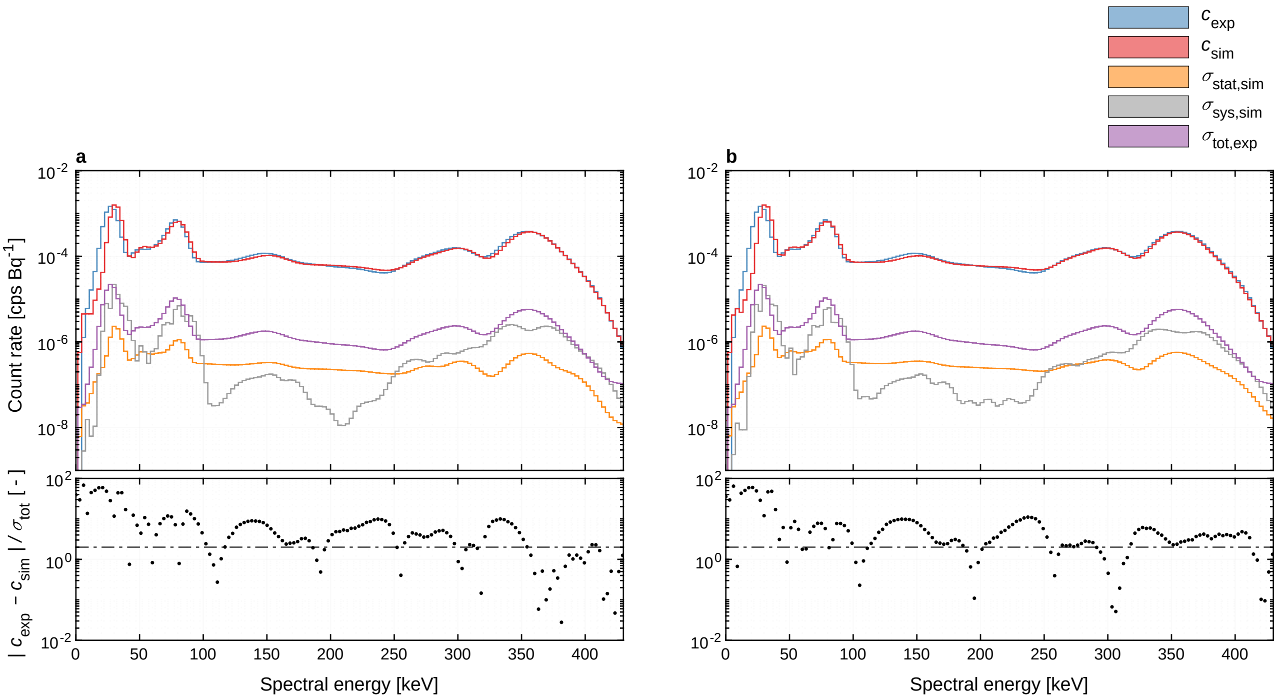

Fig. S24: **Uncertainty quantification for the ^{133}Ba spectral detector response.** The measured and simulated mean net count rates c_{exp} and c_{sim} are shown for the sum channel using a ^{133}Ba calibrated radionuclide source ($A = 2.152(32) \times 10^5$ Bq) together with the corresponding uncertainty estimates, i.e. the combined statistical and systematic measured uncertainty $\sigma_{\text{tot,exp}}$, the simulated statistical uncertainty $\sigma_{\text{stat,sim}}$ as well as the simulated systematic uncertainty $\sigma_{\text{sys,sim}}$, using 1 standard deviation values. The measurement results were presented already elsewhere [9]. Two different scintillation models have been used for the simulations: **a** Proportional scintillation model published in [9]. **b** Bayesian calibrated non-proportional scintillation model obtained by the sum mode inversion pipeline presented in this study. The normalized residual level $\mid c_{\text{exp}} - c_{\text{sim}} \mid / \sigma_{\text{tot}}$ with $\sigma_{\text{tot}} := \sqrt{\sigma^2_{\text{tot,exp}} + \sigma^2_{\text{tot,sim}}}$ for a coverage factor of 2 is marked with the horizontal dash-dotted black line in the lower subfigures. More information on the numerical computation of the uncertainty estimates can be found in Section S1.3 and [9].

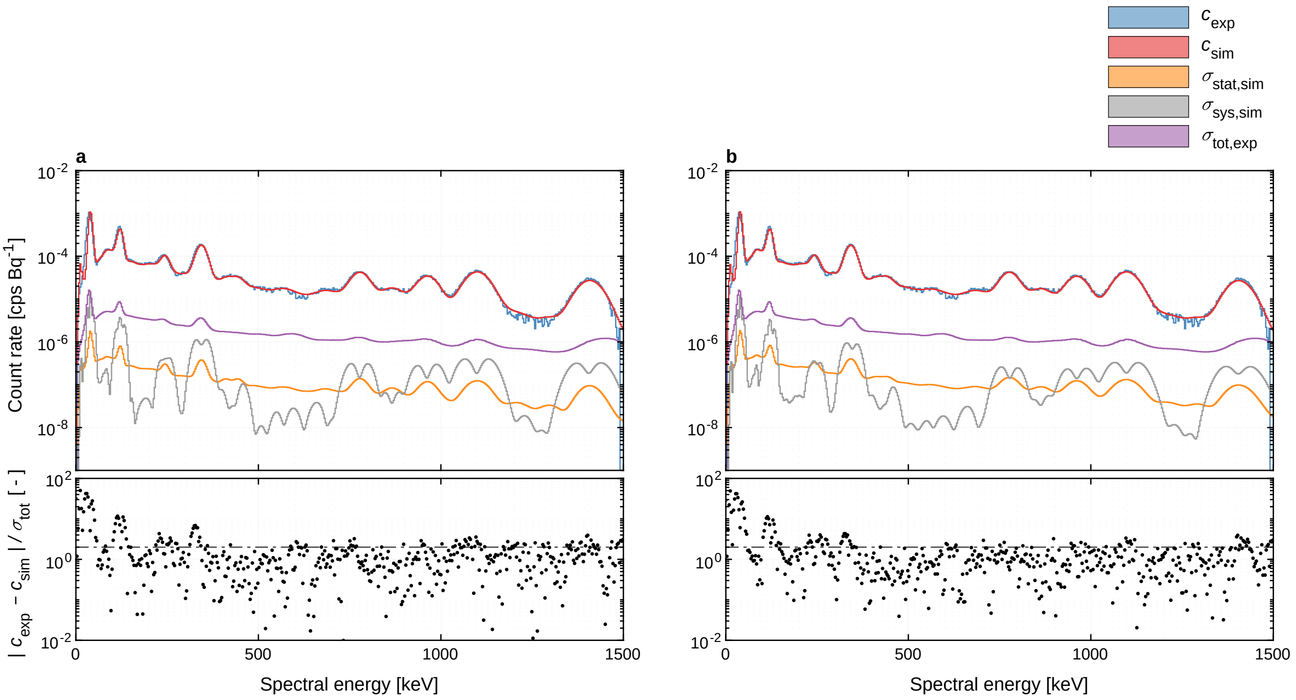

Fig. S25: **Uncertainty quantification for the ^{152}Eu spectral detector response.** The measured and simulated mean net count rates c_{exp} and c_{sim} are shown for the sum channel using a ^{152}Eu calibrated radionuclide source ($A = 1.973(30) \times 10^4$ Bq) together with the corresponding uncertainty estimates, i.e. the combined statistical and systematic measured uncertainty $\sigma_{\text{tot,exp}}$, the simulated statistical uncertainty $\sigma_{\text{stat,sim}}$ as well as the simulated systematic uncertainty $\sigma_{\text{sys,sim}}$, using 1 standard deviation values. The measurement results were presented already elsewhere [9]. Two different scintillation models have been used for the simulations: **a** Proportional scintillation model published in [9]. **b** Bayesian calibrated non-proportional scintillation model obtained by the sum mode inversion pipeline presented in this study. The normalized residual level $\mid c_{\text{exp}} - c_{\text{sim}} \mid / \sigma_{\text{tot}}$ with $\sigma_{\text{tot}} := \sqrt{\sigma^2_{\text{tot,exp}} + \sigma^2_{\text{tot,sim}}}$ for a coverage factor of 2 is marked with the horizontal dash-dotted black line in the lower subfigures. More information on the numerical computation of the uncertainty estimates can be found in Section S1.3 and [9].

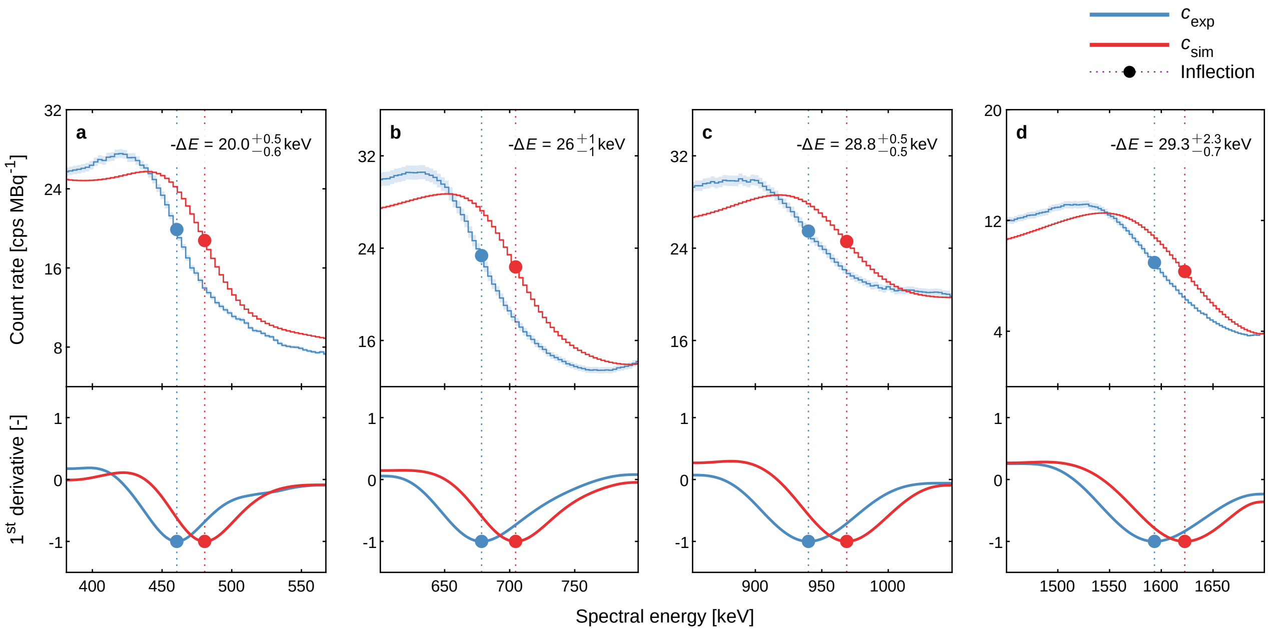

Fig. S26: **Compton edge shift analysis for the sum channel.** Here, we present the results from our Compton edge shift analysis for the sum channel. We characterize this negative spectral shift $-\Delta E$ for four different Compton edges: **a** 477.334(3) keV associated with the ^{137}Cs emission line at 661.657(3) keV. **b** 699.133(3) keV associated with the ^{88}Y emission line at 898.042(3) keV. **c** 963.419(3) keV associated with the ^{60}Co emission line at 1173.228(3) keV. **d** 1611.77(1) keV associated with the ^{88}Y emission line at 1836.063(3) keV. First, we determine the inflection points at the individual Compton edges for the measured net spectra c_{exp} and the simulated net spectra c_{sim} (proportional scintillation model) by computing the 1st derivative of the corresponding spectra using spline regression [20] (bottom panels). Note that for visualization purposes, we have normalized the first derivatives of the net count rate spectra shown in the bottom panels by their corresponding global minima. In a second step, we compute the Compton edge shift as the spectral difference between the determined inflection points for c_{exp} and c_{sim}, i.e. $-\Delta E \coloneqq c_{\text{sim}}^{\text{infl}} - c_{\text{exp}}^{\text{infl}}$ (top panels).

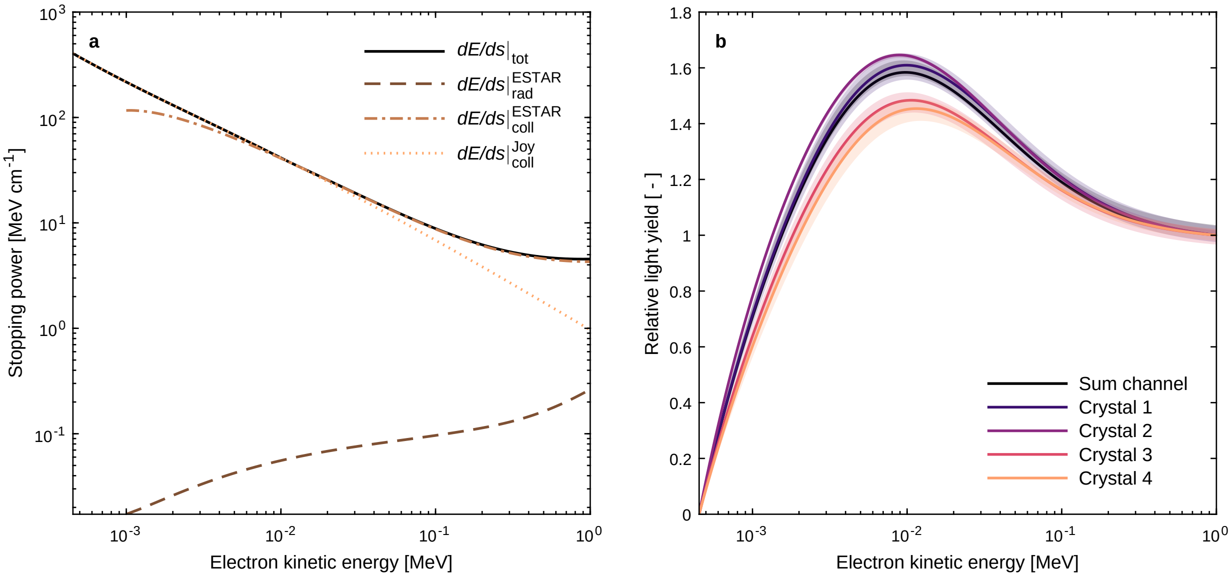

Fig. S27: **Light yield analysis.** Here, we highlight the stopping power models for electrons in NaI(Tl) alongside the resulting relative light yield curves as a function of the electron kinetic energy E_k. **a** Adopted stopping power model $dE/ds\,|_\mathrm{tot}$ based on a modified Bethe-Bloch model $dE/ds\,|^\mathrm{Joy}_\mathrm{coll}$ for collisional losses at low energies derived by Joy and Luo [23] as well as radiative and collisional losses at higher energies predicted by the `ESTAR` database [24]. **b** Relative light yield $\mathcal{L}(E_\mathrm{k})/E_\mathrm{k}$ as a function of E_k for both, the sum channel and the individual scintillation crystals associated with the sum and single mode inversion pipelines, respectively. We applied the maximum a posteriori (MAP) probability point estimates for the individual model parameters, i.e. $\eta_{e/h}$, $dE/ds\,|_\mathrm{Ons}$, $dE/ds\,|_\mathrm{Trap}$ and $dE/ds\,|_\mathrm{Birks}$, derived in the main study to compute the mean relative light yield function normalized at 1 MeV according to Eq. S30. In addition, we present 99% central credible intervals for each individual relative light yield function using the full set of posterior samples obtained by the sum and single mode inversion pipelines. A list of all material properties for NaI(Tl) used to compute the stopping power predictions as well as the resulting relative light yield functions can be found in Table S5.

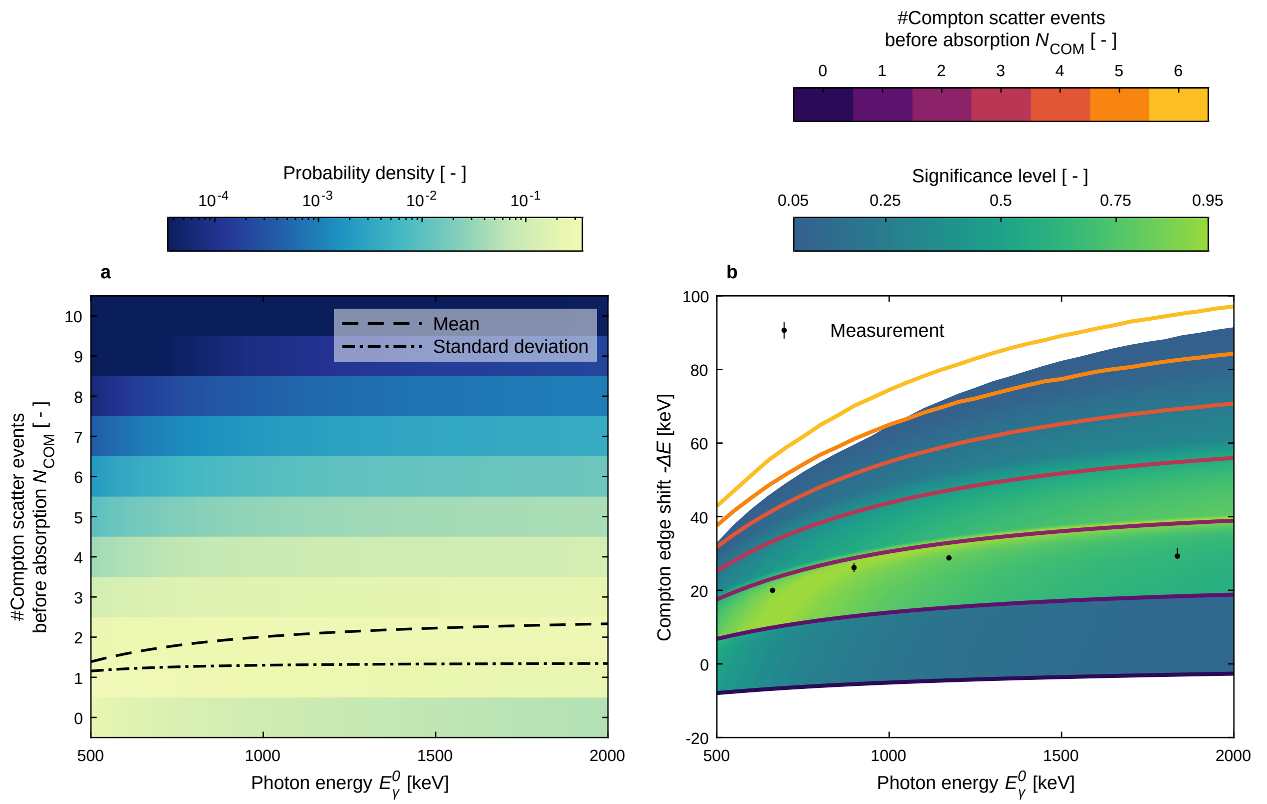

Fig. S28: **Semi-analytical model results.** Here, we present a Monte Carlo based estimate of the number of Compton scatter (COM) events before absorption (N_{COM}) together with Compton edge shift predictions using a simplified semi-analytical model derived in Section S1.4 for a $10.2\,\mathrm{cm} \times 10.2\,\mathrm{cm} \times 40.6\,\mathrm{cm}$ prismatic NaI(Tl) scintillation crystal as function of the initial photon energy E^0_γ. **a** Probability density for N_{COM} as a function of E^0_γ together with the mean and standard deviation values estimated by Monte Carlo simulations using the multi-purpose code `FLUKA` [32]. **b** Predicted median of the negative Compton edge shift $-\Delta E$ discriminated for individual number of COM events as well as the confidence interval based on the full distribution for different significance levels in the range $[0.05\,, 0.95]$ adopting the relative light yield function for the sum channel. In addition, we show the measured mean Compton edge shifts for the sum channel together with the 99% confidence intervals for four different Compton edges, i.e. $[477.334(3)\,, 699.133(3)\,, 963.419(3)\,, 1611.77(1)]\,\mathrm{keV}$ associated with the photon emission lines of the radionuclides $\{^{137}\mathrm{Cs}\,, ^{88}\mathrm{Y}\,, ^{60}\mathrm{Co}\,, ^{88}\mathrm{Y}\}$ at $[661.657(3)\,, 898.042(3)\,, 1173.228(3)\,, 1836.063(3)]\,\mathrm{keV}$, respectively.

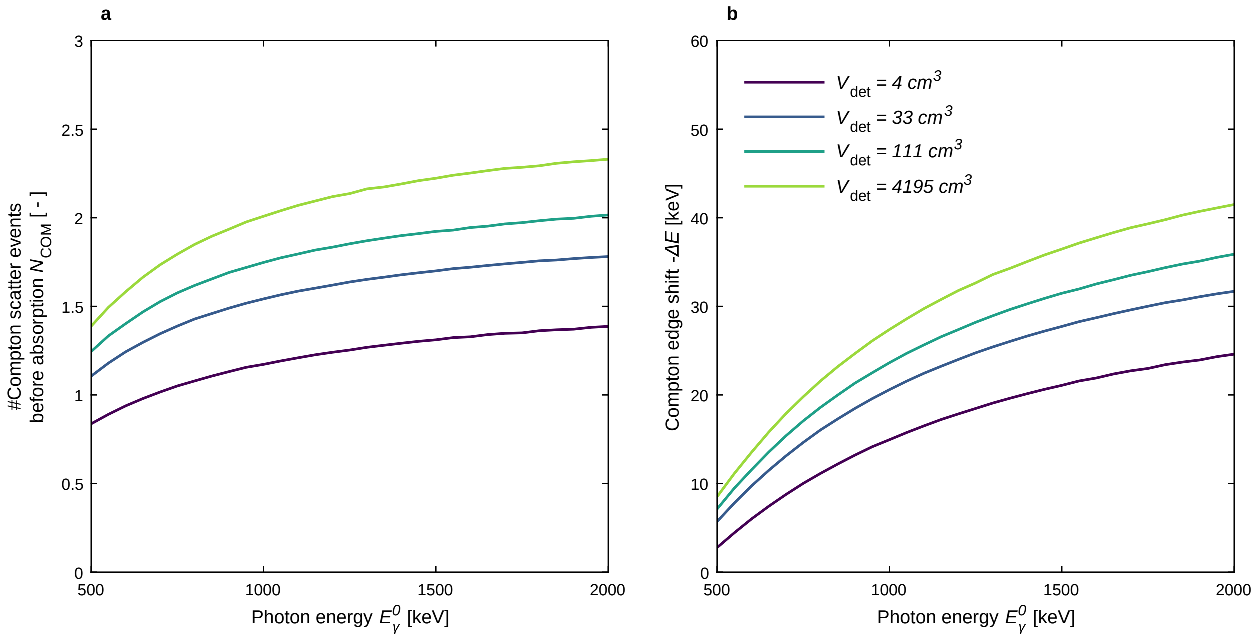

Fig. S29: **Predicted Compton edge shift for different scintillator sizes.** Here, we show Monte Carlo based estimates of the mean number of Compton scatter (COM) events before absorption (N_{COM}) together with mean Compton edge shift predictions as function of the initial photon energy E_γ^0 adopting a simplified semi-analytical model derived in Section S1.4 for four different NaI(Tl) scintillation crystals, i.e. three equilateral cylindrical crystals with characteristic lengths $[2.54\,,5.08\,,7.62]\,\mathrm{cm}$ and associated volumes (V_{det}) $[4\,,33\,,111]\,\mathrm{cm}^3$ as well as a $10.2\,\mathrm{cm} \times 10.2\,\mathrm{cm} \times 40.6\,\mathrm{cm}$ prismatic crystal with a volume of $4195\,\mathrm{cm}^3$. **a** Mean number of COM events before absorption (N_{COM}) for four different NaI(Tl) scintillation crystals as a function of E_γ^0 obtained by Monte Carlo simulations using the multi-purpose code `FLUKA` [32]. **b** Mean negative Compton edge shift $-\Delta E$ as function of the initial photon energy E_γ^0 for four different NaI(Tl) scintillation crystals predicted by the semi-analytical model derived in Section S1.4.

Supplementary Tables

Table S1: **Prior distribution summary.** This table summarizes the adopted prior distributions applied to the sum and single mode inversion pipelines for the individual model parameters, i.e. the Birks related stopping power parameter $dE/ds\ |_{\mathrm{Birks}}$, the trapping related stopping power parameter $dE/ds\ |_{\mathrm{Trap}}$, the free carrier fraction $\eta_{e/h}$ as well as the discrepancy model variance σ_{ε}^{2}. In addition, we list the consulted studies, which motivated the individual priors.

Pipeline	Variable	Prior[1]	Prior parameters[2]		Truncation	Unit	References
sum	$dE/ds\ \|_{\mathrm{Birks}}$	Uniform	$x_l = 1.5 \cdot 10^{2}$	$x_u = 4.5 \cdot 10^{2}$	–	$\mathrm{MeV\,cm^{-1}}$	[21, 22, 35]
	$dE/ds\ \|_{\mathrm{Trap}}$	Uniform	$x_l = 1.0 \cdot 10^{1}$	$x_u = 1.5 \cdot 10^{1}$	–	$\mathrm{MeV\,cm^{-1}}$	[35]
	$\eta_{e/h}$	Uniform	$x_l = 4.5 \cdot 10^{-1}$	$x_u = 6.5 \cdot 10^{-1}$	–	–	[21, 22, 35]
	σ_{ε}^{2}	Uniform	$x_l = 0$	$x_u = \sigma_{\varepsilon,\mathrm{max}}^{2}$	–	$\mathrm{cps^2\,Bq^{-2}}$	–
single	$dE/ds\ \|_{\mathrm{Birks}}$	Gaussian	$\mu = 3.23 \cdot 10^{2}$	$\sigma = 2.28 \cdot 10^{1}$	$[1.5, 4.5] \cdot 10^{2}$	$\mathrm{MeV\,cm^{-1}}$	[21, 22, 35]
	$dE/ds\ \|_{\mathrm{Trap}}$	Gaussian	$\mu = 1.43 \cdot 10^{1}$	$\sigma = 7.51 \cdot 10^{-1}$	$[1.0, 1.8] \cdot 10^{1}$	$\mathrm{MeV\,cm^{-1}}$	[35]
	$\eta_{e/h}$	Gaussian	$\mu = 5.94 \cdot 10^{-1}$	$\sigma = 6.83 \cdot 10^{-3}$	$[4.5, 6.5] \cdot 10^{-1}$	–	[21, 22, 35]
	σ_{ε}^{2}	Uniform	$x_l = 0$	$x_u = \sigma_{\varepsilon,\mathrm{max}}^{2}$	–	$\mathrm{cps^2\,Bq^{-2}}$	–

[1] We use the continuous uniform distribution $\mathcal{U}(x_l, x_u)$ with the lower and upper boundary parameters x_l and x_u to denote the uniform prior. By the Gaussian prior, we refer to the truncated univariate normal distribution $\mathcal{N}(\mu, \sigma, x_l, x_u)$ with mean μ, standard deviation σ and truncation $[x_l, x_u]$.

[2] We define the upper limit for the discrepancy model variance $\sigma_{\varepsilon,\mathrm{max}}^{2}$ as $\langle c_{\mathrm{exp}}^{2} \rangle$ with c_{exp} being the measured net count rate over the spectral Compton edge domain $\mathcal{D}_E$ for the corresponding detection channel (cf. Methods in the main study).

Table S2: **Posterior statistics summary.** This table includes posterior point and dispersion estimators for the Bayesian inverted non-proportional scintillation models obtained by the sum and single mode inversion pipelines. The listed estimators are the maximum a posteriori (MAP) probability estimate $\mathbf{x}_{\text{MAP}}$, the posterior mean $\mathbf{x}_{\text{Mean}}$ and the posterior median $\mathbf{x}_{\text{Median}}$ together with the 95% credible interval and the posterior standard deviation $\boldsymbol{\sigma}_{\mathbf{x}}$ for the parameters $\mathbf{x} := \left(dE/ds\,|_{\text{Birks}},\ dE/ds\,|_{\text{Trap}},\ \eta_{e/h},\ \sigma_\varepsilon^2\right)^\intercal$, i.e. the Birks related stopping power parameter $dE/ds\,|_{\text{Birks}}$, the trapping related stopping power parameter $dE/ds\,|_{\text{Trap}}$, the free carrier fraction $\eta_{e/h}$ as well as the discrepancy model variance σ_ε^2.

Pipeline	Variable	$\mathbf{x}_{\text{MAP}}$	$\mathbf{x}_{\text{Mean}}$	$\mathbf{x}_{\text{Median}}$	95% credible interval[1]	$\boldsymbol{\sigma}_{\mathbf{x}}$	Unit
sum	$dE/ds\,|_{\text{Birks}}$	$3.22 \cdot 10^{2}$	$3.23 \cdot 10^{2}$	$3.22 \cdot 10^{2}$	$[2.78, 3.68] \cdot 10^{2}$	$2.28 \cdot 10^{1}$	MeV cm^{-1}
	$dE/ds\,|_{\text{Trap}}$	$1.46 \cdot 10^{1}$	$1.43 \cdot 10^{1}$	$1.44 \cdot 10^{1}$	$[1.15, 1.48] \cdot 10^{1}$	$7.51 \cdot 10^{-1}$	MeV cm^{-1}
	$\eta_{e/h}$	$5.96 \cdot 10^{-1}$	$5.94 \cdot 10^{-1}$	$5.95 \cdot 10^{-1}$	$[5.79, 6.06] \cdot 10^{-1}$	$6.83 \cdot 10^{-3}$	–
	σ_ε^2	$1.24 \cdot 10^{-1}$	$1.37 \cdot 10^{-1}$	$1.34 \cdot 10^{-1}$	$[0.98, 1.92] \cdot 10^{-1}$	$2.40 \cdot 10^{-2}$	$\text{cps}^2\,\text{Bq}^{-2}$
single (crystal 1)	$dE/ds\,|_{\text{Birks}}$	$3.17 \cdot 10^{2}$	$3.17 \cdot 10^{2}$	$3.16 \cdot 10^{2}$	$[2.87, 3.49] \cdot 10^{2}$	$1.57 \cdot 10^{1}$	MeV cm^{-1}
	$dE/ds\,|_{\text{Trap}}$	$1.33 \cdot 10^{1}$	$1.32 \cdot 10^{1}$	$1.32 \cdot 10^{1}$	$[1.22, 1.43] \cdot 10^{1}$	$5.48 \cdot 10^{-1}$	MeV cm^{-1}
	$\eta_{e/h}$	$6.05 \cdot 10^{-1}$	$6.05 \cdot 10^{-1}$	$6.05 \cdot 10^{-1}$	$[5.95, 6.14] \cdot 10^{-1}$	$4.96 \cdot 10^{-3}$	–
	σ_ε^2	$2.61 \cdot 10^{-2}$	$2.83 \cdot 10^{-2}$	$2.83 \cdot 10^{-2}$	$[2.03, 3.94] \cdot 10^{-2}$	$4.92 \cdot 10^{-3}$	$\text{cps}^2\,\text{Bq}^{-2}$
single (crystal 2)	$dE/ds\,|_{\text{Birks}}$	$4.15 \cdot 10^{2}$	$4.15 \cdot 10^{2}$	$4.15 \cdot 10^{2}$	$[3.93, 4.36] \cdot 10^{2}$	$1.08 \cdot 10^{1}$	MeV cm^{-1}
	$dE/ds\,|_{\text{Trap}}$	$1.41 \cdot 10^{1}$	$1.41 \cdot 10^{1}$	$1.41 \cdot 10^{1}$	$[1.38, 1.43] \cdot 10^{1}$	$1.08 \cdot 10^{-1}$	MeV cm^{-1}
	$\eta_{e/h}$	$5.94 \cdot 10^{-1}$	$5.94 \cdot 10^{-1}$	$5.94 \cdot 10^{-1}$	$[5.88, 5.99] \cdot 10^{-1}$	$2.73 \cdot 10^{-3}$	–
	σ_ε^2	$6.92 \cdot 10^{-3}$	$7,64 \cdot 10^{-3}$	$7.50 \cdot 10^{-3}$	$[5.44, 10.68] \cdot 10^{-3}$	$1.36 \cdot 10^{-3}$	$\text{cps}^2\,\text{Bq}^{-2}$
single (crystal 3)	$dE/ds\,|_{\text{Birks}}$	$2.84 \cdot 10^{2}$	$2.81 \cdot 10^{2}$	$2.81 \cdot 10^{2}$	$[2.47, 3.19] \cdot 10^{2}$	$1.85 \cdot 10^{1}$	MeV cm^{-1}
	$dE/ds\,|_{\text{Trap}}$	$1.50 \cdot 10^{1}$	$1.53 \cdot 10^{1}$	$1.52 \cdot 10^{1}$	$[1.47, 1.61] \cdot 10^{1}$	$3.71 \cdot 10^{-1}$	MeV cm^{-1}
	$\eta_{e/h}$	$5.75 \cdot 10^{-1}$	$5.75 \cdot 10^{-1}$	$5.76 \cdot 10^{-1}$	$[5.65, 5.86] \cdot 10^{-1}$	$5.47 \cdot 10^{-3}$	–
	σ_ε^2	$2.15 \cdot 10^{-2}$	$2.31 \cdot 10^{-2}$	$2.30 \cdot 10^{-2}$	$[1.65, 3.23] \cdot 10^{-2}$	$4.08 \cdot 10^{-3}$	$\text{cps}^2\,\text{Bq}^{-2}$
single (crystal 4)	$dE/ds\,|_{\text{Birks}}$	$2.54 \cdot 10^{2}$	$2.56 \cdot 10^{2}$	$2.56 \cdot 10^{2}$	$[2.23, 2.88] \cdot 10^{2}$	$1.63 \cdot 10^{1}$	MeV cm^{-1}
	$dE/ds\,|_{\text{Trap}}$	$1.37 \cdot 10^{1}$	$1.37 \cdot 10^{1}$	$1.37 \cdot 10^{1}$	$[1.33, 1.40] \cdot 10^{1}$	$1.96 \cdot 10^{-1}$	MeV cm^{-1}
	$\eta_{e/h}$	$5.75 \cdot 10^{-1}$	$5.74 \cdot 10^{-1}$	$5.74 \cdot 10^{-1}$	$[5.66, 5.82] \cdot 10^{-1}$	$3.95 \cdot 10^{-3}$	–
	σ_ε^2	$1.05 \cdot 10^{-2}$	$1.15 \cdot 10^{-2}$	$1.14 \cdot 10^{-2}$	$[0.82, 1.60] \cdot 10^{-2}$	$2.00 \cdot 10^{-3}$	$\text{cps}^2\,\text{Bq}^{-2}$

[1] Central credible interval with a probability mass of 95%.

Table S3: **Compton edge domain sensitivity.** To investigate the sensitivity of the selected Compton edge domain $\mathcal{D}_E \coloneqq \{E : E_{\mathrm{CE}} - 3 \cdot \sigma_{\mathrm{tot}}(E_{\mathrm{CE}}) \leq E \leq E_{\mathrm{FEP}} - 2 \cdot \sigma_{\mathrm{tot}}(E_{\mathrm{FEP}})\}$ (cf. Methods in the main study) on the Bayesian inversion results, we have altered the domain size by 2.5% symmetrically with respect to the domain boundaries and performed the emulator training and Bayesian inversion computation on this new domain using the sum mode inversion pipeline. This alteration corresponds to $\approx$ 20% of the observed Compton edge shift (cf. Methods in the main study). These tables summarizes the posterior point and dispersion estimator results for these additional computations, i.e. the maximum a posteriori (MAP) probability estimate $\mathbf{x}_{\mathrm{MAP}}$, the posterior mean $\mathbf{x}_{\mathrm{Mean}}$ and the posterior median $\mathbf{x}_{\mathrm{Median}}$ together with the 95% credible interval and the posterior standard deviation $\boldsymbol{\sigma}_{\mathbf{x}}$ for the parameters $\mathbf{x} \coloneqq \left(dE/ds\,|_{\mathrm{Birks}},\ dE/ds\,|_{\mathrm{Trap}},\ \eta_{e/h},\ \sigma_\varepsilon^2\right)^\intercal$. These parameters are the Birks related stopping power parameter $dE/ds\,|_{\mathrm{Birks}}$, the trapping related stopping power parameter $dE/ds\,|_{\mathrm{Trap}}$, the free carrier fraction $\eta_{e/h}$ as well as the discrepancy model variance σ_ε^2.

(a) 2.5% decrease in $\mathcal{D}_E$

Parameter	$\mathbf{x}_{\mathrm{MAP}}$	$\mathbf{x}_{\mathrm{Mean}}$	$\mathbf{x}_{\mathrm{Median}}$	95% credible interval[1]	$\boldsymbol{\sigma}_{\mathbf{x}}$	Unit
$dE/ds\,\|_{\mathrm{Birks}}$	$3.08 \cdot 10^2$	$3.10 \cdot 10^2$	$3.08 \cdot 10^2$	$[2.79, 3.48] \cdot 10^2$	$2.14 \cdot 10^1$	$\mathrm{MeV\,cm^{-1}}$
$dE/ds\,\|_{\mathrm{Trap}}$	$1.50 \cdot 10^1$	$1.46 \cdot 10^1$	$1.47 \cdot 10^1$	$[1.33, 1.50] \cdot 10^1$	$6.24 \cdot 10^{-1}$	$\mathrm{MeV\,cm^{-1}}$
$\eta_{e/h}$	$5.93 \cdot 10^{-1}$	$5.92 \cdot 10^{-1}$	$5.92 \cdot 10^{-1}$	$[5.82, 6.01] \cdot 10^{-1}$	$5.85 \cdot 10^{-3}$	–
σ_ε^2	$1.05 \cdot 10^{-1}$	$1.12 \cdot 10^{-1}$	$1.18 \cdot 10^{-1}$	$[0.89, 1.56] \cdot 10^{-1}$	$2.08 \cdot 10^{-2}$	$\mathrm{cps^2\,Bq^{-2}}$

(b) 2.5% increase in $\mathcal{D}_E$

Parameter	$\mathbf{x}_{\mathrm{MAP}}$	$\mathbf{x}_{\mathrm{Mean}}$	$\mathbf{x}_{\mathrm{Median}}$	95% credible interval[1]	$\boldsymbol{\sigma}_{\mathbf{x}}$	Unit
$dE/ds\,\|_{\mathrm{Birks}}$	$3.34 \cdot 10^2$	$3.30 \cdot 10^2$	$3.31 \cdot 10^2$	$[2.90, 3.70] \cdot 10^2$	$2.46 \cdot 10^1$	$\mathrm{MeV\,cm^{-1}}$
$dE/ds\,\|_{\mathrm{Trap}}$	$1.46 \cdot 10^1$	$1.42 \cdot 10^1$	$1.43 \cdot 10^1$	$[1.22, 1.48] \cdot 10^1$	$8.70 \cdot 10^{-1}$	$\mathrm{MeV\,cm^{-1}}$
$\eta_{e/h}$	$5.95 \cdot 10^{-1}$	$5.94 \cdot 10^{-1}$	$5.95 \cdot 10^{-1}$	$[5.82, 6.04] \cdot 10^{-1}$	$7.10 \cdot 10^{-3}$	–
σ_ε^2	$1.42 \cdot 10^{-1}$	$1.58 \cdot 10^{-1}$	$1.54 \cdot 10^{-1}$	$[1.19, 2.01] \cdot 10^{-1}$	$2.75 \cdot 10^{-2}$	$\mathrm{cps^2\,Bq^{-2}}$

[1]Central credible interval with a probability mass of 95%.

Table S4: **Empirical model summary.** This table summarizes the adopted marginal distributions for the empirical model parameters discussed in Section S1.3 to quantify the systematic uncertainties for the Bayesian calibrated NPSM simulations. These parameters are the calibration factor D_1 as well as the empirical resolution parameters B_1^* and B_2.

Crystal	Variable	Distribution	Distribution parameters[1]		Truncation	Unit
1	D_1	Gaussian	$\mu = 3.31 \cdot 10^{-1}$	$\sigma = 3.6 \cdot 10^{-4}$	–	keV^{-1}
	B_1^*	Gaussian	$\mu = -5.71 \cdot 10^{-1}$	$\sigma = 5.5 \cdot 10^{-2}$	–	–
	B_2	Gaussian	$\mu = 6.39 \cdot 10^{-1}$	$\sigma = 1.1 \cdot 10^{-2}$	$[0, \infty)$	–
2	D_1	Gaussian	$\mu = 3.32 \cdot 10^{-1}$	$\sigma = 4.3 \cdot 10^{-4}$	–	keV^{-1}
	B_1^*	Gaussian	$\mu = -6.22 \cdot 10^{-1}$	$\sigma = 6.1 \cdot 10^{-2}$	–	–
	B_2	Gaussian	$\mu = 6.31 \cdot 10^{-1}$	$\sigma = 1.2 \cdot 10^{-2}$	$[0, \infty)$	–
3	D_1	Gaussian	$\mu = 3.35 \cdot 10^{-1}$	$\sigma = 2.1 \cdot 10^{-4}$	–	keV^{-1}
	B_1^*	Gaussian	$\mu = -6.23 \cdot 10^{-1}$	$\sigma = 6.0 \cdot 10^{-2}$	–	–
	B_2	Gaussian	$\mu = 6.54 \cdot 10^{-1}$	$\sigma = 1.1 \cdot 10^{-2}$	$[0, \infty)$	–
4	D_1	Gaussian	$\mu = 3.34 \cdot 10^{-1}$	$\sigma = 1.9 \cdot 10^{-4}$	–	keV^{-1}
	B_1^*	Gaussian	$\mu = -6.86 \cdot 10^{-1}$	$\sigma = 5.7 \cdot 10^{-2}$	–	–
	B_2	Gaussian	$\mu = 6.44 \cdot 10^{-1}$	$\sigma = 1.0 \cdot 10^{-2}$	$[0, \infty)$	–

[1]By the Gaussian distribution, we refer to the (truncated) univariate normal distribution $\mathcal{N}(\mu, \sigma, x_l, x_u)$ with mean μ, standard deviation σ and optional truncation $[x_l, x_u]$.

Table S5: Material properties for NaI(Tl) scintillator. In this table, we summarize all material properties associated with the inorganic scintillator NaI(Tl), which were used for the Compton edge shift analysis in Section S1.4. Moreover, we list the references, which were consulted to retrieve the individual numerical values.

Quantity	Symbol	Numerical value	Unit	Reference
Atomic number	Z	64	–	[24]
Mass density	ρ	3.667	$\mathrm{g\,cm^{-3}}$	[24]
Mean excitation energy	I	452	eV	[24]
Molecular weight	A	149.89424	–	[24]
Stopping power correction factor	c	2.8	–	[21, 22]

Supplementary Algorithms

Algorithm S1 COMSCW($dE/ds\,|_{\mathrm{Birks}}$, $dE/ds\,|_{\mathrm{Ons}}$, $dE/ds\,|_{\mathrm{Trap}}$, $\eta_{e/h}$)

COMSCW is a custom user-routine for the multi-purpose Monte Carlo code FLUKA [32] called at each energy deposition event in the scintillation crystal. We adapt this routine by weighting each electron or positron energy deposition event by the adopted non-proportional scintillation model (NPSM) [35]. The algorithm accounts for both continuous as well as local energy deposition events. As described in the Methods section in the main study, we set a kinetic energy threshold of 1 keV below which the electrons and positrons as well as particles generated by atomic deexcitation are no longer transported and their energy is deposited on the spot. We refer to these events as "local" energy deposition events. On the other hand, above this threshold, ionization losses are evenly distributed along the particle step [36, 37]. Hence, we call these events "continuous". The pseudo-code added below is a simplified version of the one implemented in our forward model. For more details, we kindly refer to the actual routine deposited on the ETH Research Collection repository under accession code https://doi.org/10.3929/ethz-b-000595727 [38].

---

load EventID ▷ EventID = {"continuous", "local"}
load ParticleID ▷ Particle type
load dE ▷ Deposited energy
if EventID = "continuous" AND (ParticleID = "electron" OR ParticleID = "positron") **then**
  load ds ▷ Curved particle path
  $S = dE/ds$ ▷ Estimate stopping power S
else if EventID = "local" **then**
  load E_{k} ▷ Kinetic particle energy
  if ParticleID = "electron" **then**
    S = EDEDXT(E_{k}) ▷ Call built-in stopping power function EDEDXT for electrons
  else if ParticleID = "positron" **then**
    S = PDEDXT(E_{k}) ▷ Call built-in stopping power function PDEDXT for positrons
  end if
end if
$dL = dE \times \dfrac{1-\eta_{e/h}\exp\left[-\frac{S}{dE/ds|_{\mathrm{Ons}}}\exp\left(-\frac{dE/ds|_{\mathrm{Trap}}}{S}\right)\right]}{1+\frac{S}{dE/ds|_{\mathrm{Birks}}}}$ ▷ Weight deposited energy by NPSM

Supplementary References

[1] Blatman, G. & Sudret, B. Sparse polynomial chaos expansions of vector-valued response quantities. *Proc. Int. Conf. ICOSSAR* (2013).

[2] Nagel, J. B., Rieckermann, J. & Sudret, B. Principal component analysis and sparse polynomial chaos expansions for global sensitivity analysis and model calibration: Application to urban drainage simulation. *Reliab. Eng. Syst. Saf.* **195,** 106737 (2020).

[3] Wagner, P. R., Fahrni, R., Klippel, M., Frangi, A. & Sudret, B. Bayesian calibration and sensitivity analysis of heat transfer models for fire insulation panels. *Eng. Struct.* **205,** 110063 (2020).

[4] Lüthen, N., Marelli, S. & Sudret, B. Sparse Polynomial Chaos Expansions: Literature Survey and Benchmark. *SIAM/ASA J. Uncertain. Quantif.* **9**, 593–649 (2021).

[5] Blatman, G. & Sudret, B. Adaptive sparse polynomial chaos expansion based on least angle regression. *J. Comput. Phys.* **230,** 2345–2367 (2011).

[6] Sudret, B. Global sensitivity analysis using polynomial chaos expansions. *Reliab. Eng. Syst. Saf.* **93,** 964–979 (2008).

[7] Homma, T. & Saltelli, A. Importance measures in global sensitivity analysis of nonlinear models. *Reliab. Eng. Syst. Saf.* **52,** 1–17 (1996).

[8] Sobol, I. M. Global sensitivity indices for nonlinear mathematical models and their Monte Carlo estimates. *Math. Comput. Simul.* **55,** 271–280 (2001).

[9] Breitenmoser, D., Butterweck, G., Kasprzak, M. M., Yukihara, E. G. & Mayer, S. Experimental and Simulated Spectral Gamma-Ray Response of a NaI(Tl) Scintillation Detector used in Airborne Gamma-Ray Spectrometry. *Adv. Geosci.* **57,** 89–107 (2022).

[10] Knoll, G. F. *Radiation Detection and Measurement* (John Wiley & Sons, New York, 2010).

[11] Abernethy, R. B., Benedict, R. P. & Dowdell, R. B. ASME measurement uncertainty. *J. Fluids Eng. Trans. ASME* **107,** 161–164 (1985).

[12] Pearce, A. Recommended nuclear decay data. Tech. Rep., National Physical Laboratory, Teddington (2008).

[13] Forster, R. A., Booth, T. E. & Pederson, S. P. Ten new checks to assess the statistical quality of Monte Carlo solutions in MCNP. *Proc. Int. Conf. ICRS* (1994).

[14] Sklar, A. Fonctions de repartition an dimensions et leurs marges. *Publ. inst. Stat. Univ. Paris* **8,** 229–231 (1959).

[15] Joe, H. *Dependence modeling with copulas* (CRC Press, New York, 2014).

[16] Nelsen, R. B. *An Introduction to Copulas* (Springer, Secaucus, 2006).

[17] Cherubini, R., Moschini, G., Nino, R., Policroniades, R. & Varela, A. Gamma calibration of organic scintillators. *Nucl. Instrum. Methods Phys. Res. A* **281,** 349–352 (1989).

[18] Świderski, L. et al. Measurement of Compton edge position in low-Z scintillators. *Radiat. Meas.* **45,** 605–607 (2010).

[19] Mauritzson, N. et al. GEANT4-based calibration of an organic liquid scintillator. *Nucl. Instrum. Methods Phys. Res. A* **1023,** 165962 (2022).

[20] Reinsch, C. H. Smoothing by spline functions. *Numer. Math.* **10,** 177–183 (1967).

[21] Payne, S. A. et al. Nonproportionality of scintillator detectors: Theory and experiment. *IEEE Trans. Nucl. Sci.* **56,** 2506–2512 (2009).

[22] Payne, S. A. et al. Nonproportionality of scintillator detectors: Theory and experiment. II. *IEEE Trans. Nucl. Sci.* **58,** 3392–3402 (2011).

[23] Joy, D. C. & Luo, S. An empirical stopping power relationship for low-energy electrons. *Scanning* **11,** 176–180 (1989).

[24] Berger, M., Coursey, J., Zucker, M. & Chang, J. ESTAR, PSTAR, and ASTAR: Computer Programs for Calculating Stopping-Power and Range Tables for Electrons, Protons, and Helium Ions (version 2.0.1). URL http://physics.nist.gov/Star (2017).

[25] Wayne, L. R., Heindl, W. A., Hink, P. L. & Rothschild, R. E. Response of NaI(Tl) to X-rays and electrons. *Nucl. Instrum. Methods Phys. Res. A* **411,** 351–364 (1998).

[26] Khodyuk, I. V., Rodnyi, P. A. & Dorenbos, P. Nonproportional scintillation response of NaI:Tl to low energy x-ray photons and electrons. *J. Appl. Phys.* **107,** 113513 (2010).

[27] Rooney, B. D. & Valentine, J. D. Scintillator light yield nonproportionality: Calculating photon response using measured electron response. *IEEE Trans. Nucl. Sci.* **44,** 509–516 (1997).

[28] Hull, G. et al. Measurements of NaI(Tl) electron response: Comparison of different samples. *IEEE Trans. Nucl. Sci.* **56,** 331–336 (2009).

[29] Ribberfors, R. Relationship of the relativistic Compton cross section to the momentum distribution of bound electron states. *Phys. Rev. B* **12,** 2074 (1975).

[30] Brusa, D., Stutz, G., Riveros, J., Fernández-Varea, J. & Salvat, F. Fast sampling algorithm for the simulation of photon Compton scattering. *Nucl. Instrum. Methods Phys. Res. A* **379,** 167–175 (1996).

[31] Klein, O. & Nishina, Y. Über die Streuung von Strahlung durch freie Elektronen nach der neuen relativistischen Quantendynamik von Dirac. *Z. Phys.* **52,** 853–868 (1929).

[32] Ahdida, C. et al. New Capabilities of the FLUKA Multi-Purpose Code. *Front. Phys.* **9,** 788253 (2022).

[33] Cano-Ott, D. et al. Monte Carlo simulation of the response of a large NaI(Tl)total absorption spectrometer for β-decay studies. *Nucl. Instrum. Methods Phys. Res. A* **430,** 333–347 (1999).

[34] Goodman, J. & Weare, J. Ensemble samplers with affine invariance. *Commun. Appl. Math. Comput.* **5,** 65–80 (2010).

[35] Payne, S. A., Hunter, S., Ahle, L., Cherepy, N. J. & Swanberg, E. Nonproportionality of scintillator detectors. III. Temperature dependence studies. *IEEE Trans. Nucl. Sci.* **61,** 2771–2777 (2014).

[36] Ferrari, A., Sala, P. R., Guaraldi, R. & Padoani, F. An improved multiple scattering model for charged particle transport. *Nucl. Instrum. Methods Phys. Res. B* **71,** 412–426 (1992).

[37] Battistoni, G. et al. Overview of the FLUKA code. *Ann. Nucl. Energy* **82,** 10–18 (2015).

[38] Breitenmoser, D., Cerutti, F., Butterweck, G., Kasprzak, M. M. & Mayer, S. FLUKA user routines for non-proportional scintillation simulations. ETH Research Collection https://doi.org/10.3929/ETHZ-B-000595727 (2023).
